# How robust are estimates of key parameters in standard viral dynamic models?

**DOI:** 10.1371/journal.pcbi.1011437

**Published:** 2024-04-16

**Authors:** Carolin Zitzmann, Ruian Ke, Ruy M. Ribeiro, Alan S. Perelson

**Affiliations:** Theoretical Biology and Biophysics Group, Theoretical Division, Los Alamos National Laboratory, Los Alamos, New Mexico; University of Zurich, SWITZERLAND

## Abstract

Mathematical models of viral infection have been developed, fitted to data, and provide insight into disease pathogenesis for multiple agents that cause chronic infection, including HIV, hepatitis C, and B virus. However, for agents that cause acute infections or during the acute stage of agents that cause chronic infections, viral load data are often collected after symptoms develop, usually around or after the peak viral load. Consequently, we frequently lack data in the initial phase of viral growth, i.e., when pre-symptomatic transmission events occur. Missing data may make estimating the time of infection, the infectious period, and parameters in viral dynamic models, such as the cell infection rate, difficult. However, having extra information, such as the average time to peak viral load, may improve the robustness of the estimation. Here, we evaluated the robustness of estimates of key model parameters when viral load data prior to the viral load peak is missing, when we know the values of some parameters and/or the time from infection to peak viral load. Although estimates of the time of infection are sensitive to the quality and amount of available data, particularly pre-peak, other parameters important in understanding disease pathogenesis, such as the loss rate of infected cells, are less sensitive. Viral infectivity and the viral production rate are key parameters affecting the robustness of data fits. Fixing their values to literature values can help estimate the remaining model parameters when pre-peak data is missing or limited. We find a lack of data in the pre-peak growth phase underestimates the time to peak viral load by several days, leading to a shorter predicted growth phase. On the other hand, knowing the time of infection (e.g., from epidemiological data) and fixing it results in good estimates of dynamical parameters even in the absence of early data. While we provide ways to approximate model parameters in the absence of early viral load data, our results also suggest that these data, when available, are needed to estimate model parameters more precisely.

## Author summary

In a typical acute infection, the viral load initially surges rapidly, peaks, and subsequently declines. Data collection typically begins after symptoms appear, usually around or after the peak viral load, and thus missing the critical early phase of viral growth, when pre-symptomatic transmission occurs. In the present study, we evaluate the impact of missing pre-peak data on estimating key parameters in viral dynamic models. This lack of early data complicates estimating crucial parameters in viral dynamic models, such as the time of infection, leading to shorter predicted growth phases. We found that while model parameter estimates of the time of infection are sensitive to data quantity, parameters like the loss rate of infected cells are less affected. Conversely, knowing the time of infection and fixing it can improve the robust estimation of dynamical parameter estimates even without early data. However, incorporating additional information, such as the average time to peak viral load, can enhance parameter estimation robustness. Fixing certain parameters, such as viral infectivity and production rate, to literature values can improve parameter estimation robustness when pre-peak data is limited.

## Introduction

**Fig 1:**
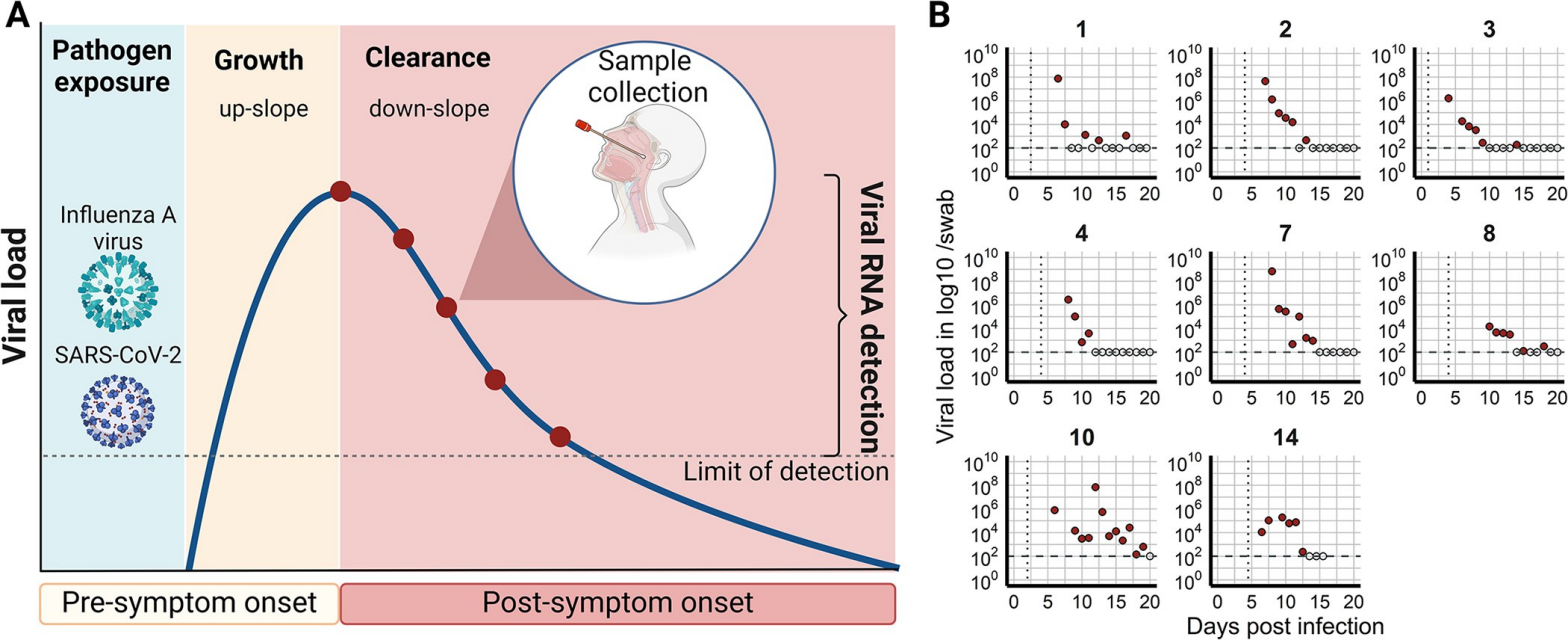
A) Typical course of an acute infection, e.g., influenza A virus or SARS-CoV-2, where the viral load initially increases (growth, up-slope), peaks, and decreases (clearance, down-slope). Viral load data is usually collected post-symptom onset (red dots). B) Example of SARS-CoV-2 data collection that starts 5 to 7 days post infection (from Wolfel et al. [3]). Filled red circles are viral load measurements above the limit of detection (horizontal dashed line), non-filled circles are below the limit of detection. The vertical dotted line represents the time of symptom onset as reported in [26]. [Created with BioRender.com].

In a typical acute infection, the viral load initially increases rapidly, reaches a peak, and then declines (Fig 1A). The same pattern is seen in infections, such as HIV or hepatitis C, which can progress from acute to chronic, where the decline does not necessarily lead to the elimination of the virus. The viral load frequently correlates with a person’s infectiousness and, thus, the probability of viral transmission [1–8]. Understanding the viral dynamics throughout the course of infection, including prior to the viral peak, is critical to understanding viral transmission [9–11]. However, viral load data is often obtained from settings where infected individuals are tested and identified days or weeks after symptoms develop [3,12–14]. In the case of influenza A virus, SARS-CoV-2, and respiratory syncytial virus, symptom onset is usually around the peak viral load and corresponds to the time when a person is highly contagious [1,2,15–20]. Consequently, as viral load measurements in infected individuals are often collected post-symptom onset, we typically lack data collected during the viral growth phase and, thus, pre-peak viral load (Fig 1B). Additionally, the exact time of pathogen exposure is often unknown, or estimates of infection times are often based on incomplete data. Knowing the time of infection and the time to peak viral load can be used to determine the duration of quarantine, help in contact tracing, and determine the period high-risk infected individuals should be monitored for symptoms. Furthermore, understanding the dynamics of viral infection may also help in the timing of antiviral treatment to clear the infection, prevent viral rebound or emerging resistance, and decrease shedding duration [21–25].

Mathematical modeling has a long history in studying the dynamics of many viruses causing acute and chronic infections, including West Nile virus [27], dengue [28–32], Zika [33–35], respiratory syncytial virus [36,37], influenza [25,38–42], SARS-CoV-2 [1,12,16,22–24,43–50], hepatitis C virus [51–58], hepatitis B virus [59–66] and HIV [67–76]. Interestingly, the model structure to study these varied infections is very similar. Those models were used to understand the fundamentals of viral infections, study the role of the immune system, and antiviral treatment efficacy. Furthermore, mathematical modeling has been a valuable tool in aiding decision-making in the SARS-CoV-2 pandemic [77–81].

The accuracy of model predictions depends on model complexity, chosen initial conditions, parameter estimation algorithm, and, most importantly, the quantity and quality of experimental measurements and clinical and epidemiological data. The impact of these issues in parameter estimation of viral dynamic models has been analyzed in previous studies [82–86]. In addition, other studies also addressed issues of structural and practical identifiability of model parameters [86–88].

Here, we analyze the effect of having different amounts of data early on (as in previous studies [50,82,85,86,89,90]), the effect of knowing some parameters, and the effect of knowing the time of infection. We then quantify how this extra information impacts parameter estimates. We are not interested in any particular infection since the early behavior (in Fig 1) is similar across many different infections, and the models used also have very similar structure; our approach should be valid for different infections. However, to be concrete, we use, as an example, data from infection with SARS-CoV-2, since this has become one of the infections with the most data. In particular, we use a data set with frequent measurements throughout, before, and after the peak of viral load [13,14]. We found that the cell infection and virus production rate are crucial parameters in viral dynamic models needed to robustly estimate the dynamics of the viral growth phase. If data is missing in the first few days post-infection, knowing those parameters (e.g., from prior studies) led to similar predictions of viral load as having frequent viral load measurements in the pre-peak growth phase. Alternatively, knowledge of the time of infection (e.g., from epidemiological evidence) or assuming a given duration until peak viral load is attained (e.g., in SARS-CoV-2 assuming the median of 5 days [91–104]) represent promising alternatives to obtain consistent parameter estimates. Nevertheless, frequent viral load measurements at the early stages of infection are crucial for obtaining more precise estimates of model parameters and the overall viral trajectory within individuals.

## Methods

### Mathematical model

A mathematical model often used to study viral infections is the target cell limited model (TCLM) with an eclipse phase, which was introduced to study influenza infection [25]. This model has been used to study various acute infections, such as Zika, dengue, influenza A, West Nile virus, Ebola, and SARS-CoV-2 [1,25,27,35,43,58], due to its simplicity and small number of parameters.

The TCLM describes the dynamics of target cells, i.e., cells susceptible to infection, *T*, infected cells in the eclipse phase that are not yet virus-producing, *E*, virus-producing infected cells, *I*, and virus, *V*. The TCLM has also been augmented by including an innate immune response that has provided a better description of influenza and SARS-CoV-2 infection dynamics [1,25]. In this model, we include a population of cells that are refractory to infection, *R*, and call the model the refractory cell model (RCM). Refractory cells are in an antiviral state induced by the innate immune response mediated by type I and type III interferons [105–107]. The following system of ordinary differential equations (ODEs) gives the dynamics of the five populations of the RCM:

$$\frac{dT}{dt}=-\beta TV-\varphi IT+\rho R,$$ (1)

$$\frac{dR}{dt}=\varphi IT-\rho R,$$

$$\frac{dE}{dt}=\beta TV-kE,$$

$$\frac{dI}{dt}=kE-\delta I,$$

$$\frac{dV}{dt}=\pi I-cV.$$

**Fig 2:**
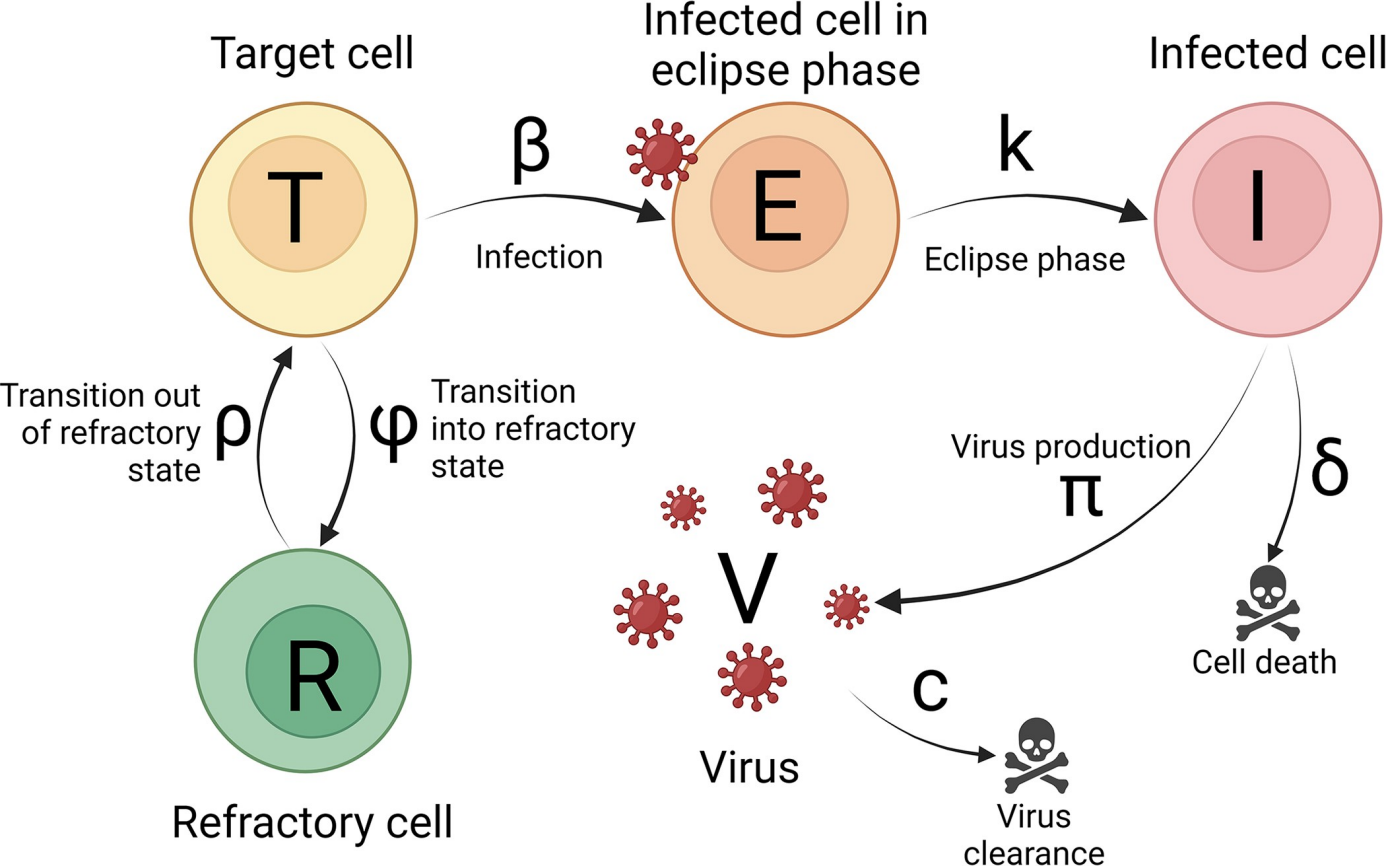
Schematic illustration of the refractory cell model. A susceptible target cell, *T*, is infected by virus, *V*, with the infection rate constant *β*. Infected cells in the eclipse phase, *E*, become actively virus-producing cells, *I*, with the transition rate constant *k*. Infected cells, *I*, produce virus with production rate constant *π* or die with degradation rate *δ*. Virus is cleared with clearance rate *c*. In the refractory cell model, in addition we also account for the innate immune response, which turns susceptible cells into refractory cells,*R*, with constant rate *φ*, which are in an antiviral state and refractory to infection. However, refractory cells can become susceptible to infection with constant rate *ρ*. [Created with BioRender.com].

The equations of the TCLM are similar and to obtain them we remove the *dR*/*dt* equation, and the terms *φIT* and *ρR* in the *dT/dt* equation (see equation S1 in S1 Text). In the model, target cells, *T*, become infected by virus with rate constant *β* and then enter the eclipse phase, *E*, which lasts for an average duration 1/*k* during which time they produce no virus. At the end of the eclipse phase, cells become productively infected cells, *I*, produce virus with rate constant *π* and die with rate constant *δ*. Note that the average infected cell lifespan is $\frac{1}{k}+\frac{1}{\delta }$. Finally, virus, *V*, is cleared with first-order rate constant *c* (Fig 2). In infections such as HIV, where virus and infected cells are sampled in blood, *π* represents the average rate of viral production by an infected cell. However, when the virus is isolated from swabs, such as in respiratory infections (e.g., SARS-CoV-2), *π* is the viral production rate multiplied by the fraction of virus sampled on a swab contained in 1 mL of viral transport fluid as described in the supporting information in Ke et al. [1].

Infection induces the release of interferons that may establish an antiviral state in non-infected target cells. For simplicity, we do not explicitly include interferons but model their concentration as proportional to the number of infected cells (for more details, see supporting information in [1]). We model the effect of interferon by assuming target cells enter the refractory state with rate *φIT*, and leave it with a constant first-order rate constant *ρ*, making them susceptible to infection again (Fig 2).

Here, for concreteness, we will focus on SARS-CoV-2 infection. Consistent with our previous work [1], we fixed the initial target cell population at the time of infection (*t*_*inf*_) to *T*(*t*_*inf*_) = 8×10^7^ cells. The initial target cell population is based on the estimated number of epithelial cells in the upper respiratory cell tract (4×10^8^ cells), with 20% of them expressing angiotensin-converting enzyme 2 (ACE2), the entry receptor used by SARS-CoV-2 [25,108]. Note that in these models, the initial target cell population and the virus production rate, *π*, are correlated and only identifiable as a product. Thus, an increase or decrease in target cells decreases or increases the virus production proportionally [25,39,86,87,109,110].

Additionally, we assumed the initial refractory cell population *R*(*t*_*inf*_) = 0 cells. The virus concentration that initiates infection is hard to estimate. Thus, as has been done previously [111], we set *V*(*t*_*inf*_) = 0 and started the growth phase of the infection with one infected cell in the eclipse phase *E*(*t*_*inf*_) = 1 [1], which was the value that led to the best model fit according to our sensitivity analysis (Table A in S2 Text). Note that it is possible that there is a variable time of stochastic rounds of infection before the virus starts to increase, and we are not including that phenomenon in our model (see [89] for details of this process in HIV). *In vitro* experiments have shown that it usually takes 4–8 hours before an infected cell starts to produce SARS-CoV-2 [22,25,112], yielding a rate of transition out of the eclipse phase of *k* = 4 *d*^−1^. Further, we fixed the virus clearance rate *c* = 10 *d*^−1^ [22] (for more details, see supporting information in [1]).

For the above models, the basic reproductive number (*R*_0_) is given by

$$R_{0}=\frac{\beta \pi T(t_{inf})}{c\delta },$$ (2)

which corresponds to the average number of cells infected by one single infected cell at the start of infection.

### Parameter estimation, model selection, and model analysis

Fitting the model to log10-transformed data was implemented using the non-linear mixed effects modeling framework in *Monolix* (lixoft.com) version 2023R1 (see S2 Text for the Monolix function used). In this approach, we assume that a model parameter *η*_*i*_ is drawn from a distribution with a fixed part *θ*, which is the median value of the parameter in the population, and a random term (for individual variability) *ϕ*_*i*_, which is assumed to be normally distributed with zero mean and standard deviation *σ*_*θ*_. Unless otherwise specified, we assumed that all parameters follow a lognormal distribution. Note that for fixed model parameters, random effects were not included. To minimize the negative log-likelihood, Monolix uses the SAEM (Stochastic Approximation Expectation-Maximization) algorithm with a Markov Chain Monte Carlo (MCMC) procedure to sample the conditional distribution of the parameters. We used all default settings and a constant error model. The 95% confidence intervals were calculated with the R package *Rsmlx* [113] using Monolix’s Fisher Information Matrix. We conducted at least 100 different parameter estimation rounds using Monolix’s R-functions with randomly chosen initial parameter values uniformly distributed within the following ranges: *t*_*inf*_∈[−8,−5] days, log_10_(*β*)∈[−8,−5] mL/RNA copies/days, *δ*∈[0.1,3] 1/day, *π*∈[1,100] RNA copies/mL/day, log_10_(*φ*)∈[−8,−4] 1/cell/day, and log_10_(*ρ*)∈[−3,−1] 1/ day. Note that these ranges do not correspond to restrictions on the parameter space; rather, each set corresponds to the ranges sampled from the initial parameters guesses for a full fitting run. Thus, the estimated parameters could lie inside or outside these ranges. Indeed, one would expect that for very simple fitting problems (simple model and large, well-behaved data sets, e.g., with a well-behaved global minimum), all of these fits would lead to the exact same parameter estimates. From the more than 100 different parameter estimation rounds (i.e., 100 randomly chosen sets of initial parameter values), the best model fit was selected for each data set by comparing the negative log-likelihood (-LL) and root-mean-square error (RMSE). We analyzed practical identifiability using the profile likelihood estimation (PLE) method for each fitted model parameter using Monolix’s R-functions (see S2 Text) [114–117]. A model parameter is identifiable if its likelihood profile is finite (crosses the confidence level) in a U-shape [117].

### Data and subsets of data

We used published data from the National Basketball Association (NBA), where unvaccinated individuals were regularly tested during the NBA tournament in 2020 and 2021 [13,14]. The data is based on a combination of anterior nares and oropharyngeal swabs tested using RT-qPCR. The resulting Ct values for each individual were converted to viral RNA (genome equivalents per mL) as described by Kissler et al. [13,14].

We chose 25 unvaccinated individuals from this cohort (9 from [13] and 16 from [14]) with frequent viral load measurements, i.e., individuals with four or more viral load measurements above the limit of detection [LOD ≥ log10(2.658) RNA copies/mL], representing the entire course of infection (viral load up-slope, peak, and down-slope) and no viral load above detection more than 14 days pre- and post-peak. For more information about this selection process, see S2 Text. We excluded vaccinated individuals since breakthrough infections involve different immune mechanisms, such as the presence of neutralizing antibodies and immune memory that may lead to quicker responses against the infection as compared to in unvaccinated individuals.

On average (± standard deviation), the 25 selected individuals had 9.8 ± 3.8 viral load measurements above the LOD with 3.1 ± 1.6 data points obtained during the up-slope, one measurement representing the observed peak viral load, and 5.7 ± 3.0 measurements obtained during the post-peak down-slope. The time of peak viral load was defined in the original studies as *t* = 0 [13,14] and, thus, the time of infection, *t*_inf_, is negative and denotes the number of days before the viral peak that infection was estimated to have taken place.

We first fitted the viral dynamic models to all the viral load measurements and then compared estimated model parameters and model predictions to different subsets of the complete data set, where we included only measurements started 3, 5, and 7 days post-infection or included only post-peak viral loads (Figs C, D, E, and F in S2 Text). We are interested in how well these smaller data sets re-create the parameter estimates we obtained when fitting the full data set, which we take as the ground truth for our purposes.

To measure how well the smaller data sets allow parameter estimates that describe the full data set, we calculate the root-mean-square error (RMSE)

$$RMSE=\sqrt{\frac{\sum_{i=1}^{n}{\left(y_{i}-{\hat{y}}_{i}\right)}^{2}}{n}},$$ (3)

where *y*_*i*_ are the log10 of actual measurements and ${\hat{y}}_{i}$ are log10 model predictions at time *i* using parameters estimated with the smaller data set but for the full course of infection with all *n* non-censored data points, i.e., viral load measurements above the detection limit (LOD). Note that we are not using the RMSE to fit the data, only to calculate how close the viral loads in the full data set are to those from a model simulation generated using parameters estimated with a subset of the data.

## Results

### SARS-CoV-2 dynamics: The course of infection

**Fig 3:**
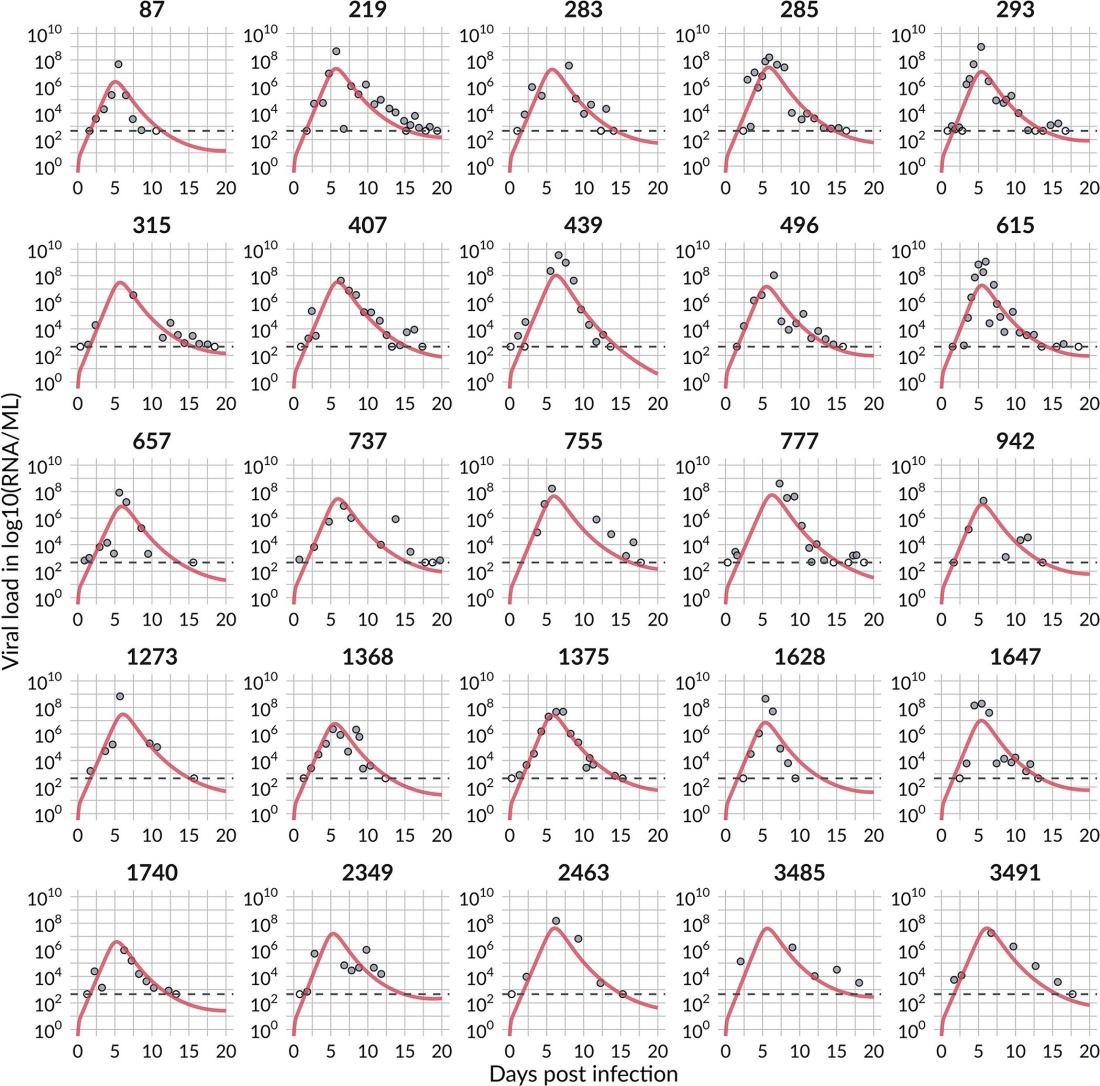
The best RCM fit to viral load measurements of the 25 selected individuals. Filled circles are measurement points; non-filled circles are censored and below the detection limit (dotted grey line). We fixed population parameters (without allowing random effects) for the initial target cell population, *T*_0_ = 8×10^7^ cells, initial eclipse cell population, *E*_0_ = 1 cell, transition rate out of the eclipse phase, *k* = 4 /day, and the virus clearance rate, *c* = 10 /day (see Table 1 and Table B in S2 Text for individual parameters, S1 Data for viral load measurements and model predictions).

The RCM fits the entire course of infection of the 25 selected individuals and describes the initial viral growth and subsequent virus clearance (see Fig A in S2 Text for the best fit with peak viral load *t* = 0 and Fig 3 for the best fit with *t*_*inf*_ shifted to zero).

**Fig 4:**
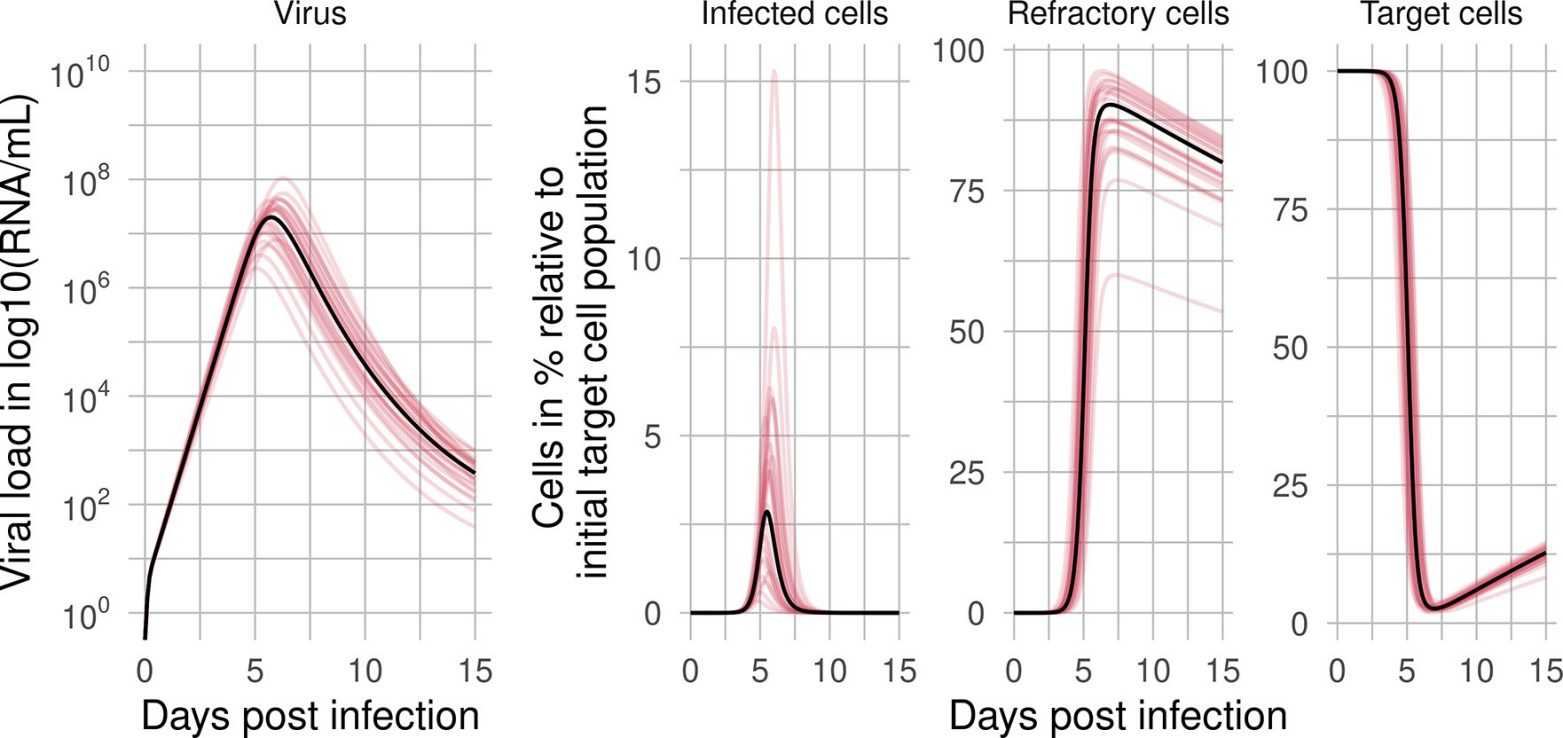
Virus and cell dynamics. Predicted dynamics of virus, infected cells, refractory cells, and target cells using the population parameters (black line) and individual parameters (colored lines) throughout the course of infection predicted by the RCM using the best-fit population parameter estimates (see S1 Data for model predictions).

We estimated the time of infection to be a median of -6.4 days from the observed viral load peak, ranging individually from -9.8 to -5.3 days (Fig A and Table B in S2 Text). This is consistent with the findings in a human challenge study [118], where the viral load peak measured with nasal swabs occurred 6.2 days after infection and ranged between 3 and 9 days. We note that the model predicted time to the peak is not necessarily the same as the time to the observed peak viral load in the data. In some individuals, the model predicts an earlier peak viral load than the observed one, e.g., the predicted viral load of individual 2349 peaks four days before the measured one, probably due to the data not showing a well-defined peak (Fig 3 and Fig A in S2 Text). The model predicts a median time to peak of 5.7 days (Fig 4), i.e., 0.7 days before the observed peak, due to a rapid increase in viral load early post-infection in some individuals. The model also predicts another 9.1 days to clear the infection (from peak viral load to the LOD), with a predicted infection duration of around 15 days. Note that the time of exposure to SARS-CoV-2 was not reported in [13,14]. Nevertheless, the last negative (below the LOD) measurement (5.8 ± 2 days pre-peak viral load) and the first positive measurement above the LOD (4.1 ± 1.7 days pre-peak viral load) were reported. Thus, our model result for the predicted time of infection is consistent with these observations.

In combination with the eclipse phase duration, 1/*k*, the average lifespan of infected cells is $\frac{1}{k}+\frac{1}{\delta }$. Using the estimated values of *k* and *δ*, we find the average lifespan of an infected cell is 0.64 days or 15 h. The within-host reproductive number *R*_0_ was, on average 5 (Table 1).

**Table 1:** Parameters in the RCM viral dynamic model and their estimated population values.

*Parameter*	*Description*	*RCM Population estimate [95% CI]*	*Unit*	*Reference*
*T*(*t*_*inf*_)	Initial target cell population	***8* × *10*** ^***7***^	Cells	[1,25,108]
*E*(*t*_*inf*_)	Initial number of infected cells in the eclipse phase population	***1***	Cells	[1] *and Table A in S2 Text*
*t*_*inf*_	Infection time	-6.4 [-6.7, -6.1]	Days	
*β*	Cell infection rate	1.07 × 10^−8^ [9.12 × 10^−9^, 1.26 × 10^−8^]	mL/RNA copies/day	
*k*	Transition rate out of the eclipse phase	***4***	1/day	[1,22,25]
*π*	Virus production rate	151 [131, 174]	RNA copies/mL/day	
*δ*	Death rate of infected cells	2.58 [2.52, 2.64]	1/day	
*c*	Virus clearance rate	***10***	1/day	[1,22]
*φ*	Target to refractory cell conversion rate constant	1.82 × 10^−6^ [1.25 × 10^−6^, 2.69 × 10^−6^]	1/cell/day	
*ρ*	Refractory to target cell conversion rate	0.016 [0.014, 0.019]	1/day	
*R*_0_	Basic reproductive number	5.0		
RMSE	Root mean squared error Sum over all individuals: Averaged over individuals:	24.3 0.97		
-LL BICc	negative log likelihood: corrected Bayesian Information Criterion:	921.3 980.8		

Values in bold were fixed.

**Fig 5:**
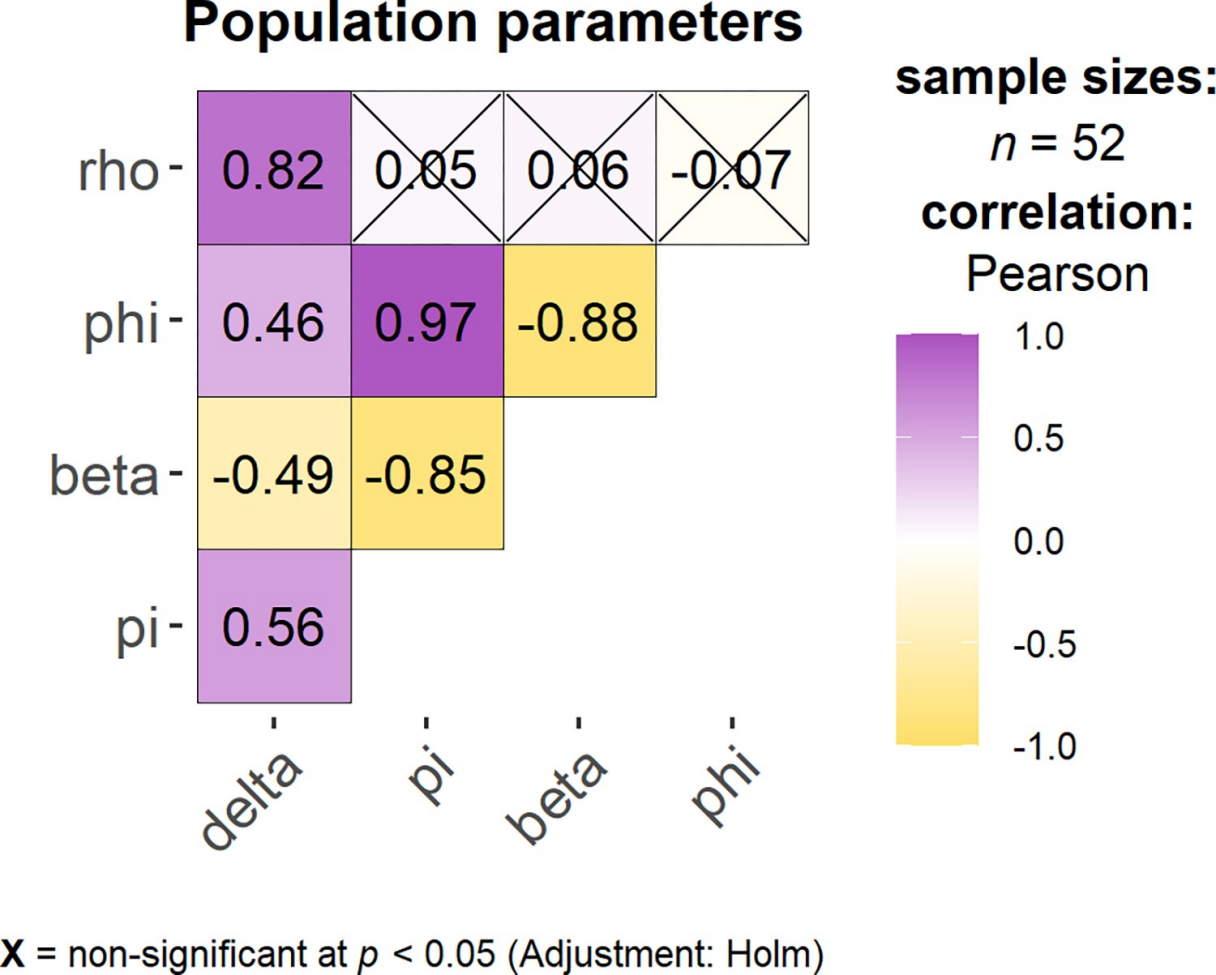
Correlation of population parameters in the refractory cell model. The sample size gives the number of fits that fit the model equally well in the range of min (-LL) to min(-LL)+2. Correlations that are crossed are non-significant (p-value > 0.05). The plot has been generated with ggstatsplot [doi:10.21105/joss.03167].

The infected cell population peaks at around the same time as the viral load (Fig 4). Target cells start to decline substantially 3 to 4 dpi until less than 3% of target cells are left at around 7 dpi when refractory cells reach their maximum of 81% of the initial susceptible target cells (Fig 4). To further explore these fits, we performed a correlation analysis of the population parameters obtained from fits with a negative log-likelihood (-LL) in the range of 2 units from the best fit [that is, min(-LL) to min(-LL) + 2]. We found more than 50 fits with a -LL in the defined range with several model parameters significantly correlated (Fig 5). For example, the cell infection rate constant (*β*) and the virus production rate (*π*) are negatively correlated, as has been seen before [40]. The transition rate of susceptible cells into refractory cells (*φ*) is positively correlated with *π* but negatively correlated with *β*. Thus, the faster the estimated rate cells transition into the refractory state, the lower the estimated cell infection rate and the higher the estimated virus production rate. Furthermore, the transition rate of refractory cells back into susceptible cells (*ρ*) is positively correlated with the loss rate of infected cells (*δ*). Note that when we included these correlations in the model fitting, there was an increase in the BICc (991 with correlations compared to 981 without). Thus, we did not include the correlations in further analyses. We further assessed model identifiability by performing a profile likelihood analysis (PLE) for every estimated model parameter. The PLE [117] showed that all estimated parameters were practically identifiable (Figs G, H, I, J, K, and L in S2 Text). Parameter identifiability for similar models was done in detail previously [82,86,87,90,112,119], and we do not explore it further here.

### Missing data in the pre-peak growth phase

In most human studies, data is not collected starting at the time of infection but rather starting at the time of or later than the onset of symptoms [16]. To understand the effect of not having early data, we constructed different data sets, with varying numbers of viral load measurements during the viral up-slope, to study the robustness (i.e., consistency) of estimated model parameters to missing data by comparison with the results obtained by fitting the full data set presented above. Based on the estimate of *t*_*inf*_ for each individual, data sets were constructed starting i) 3 days post-infection (dpi), yielding on average 2.1 ± 1.6 pre-peak measurements, ii) 5 dpi, yielding on average 0.7 ± 0.9 pre-peak measurements, and iii) 7 dpi yielding on average 0.1 ± 0.6 pre-peak measurements. In the 3, 5, and 7-dpi data sets, pre-peak viral load measurements were available from 21, 12, and 1 individual, respectively (for figures, see Figs C, D, E, and F in S2 Text). Note that the 7-dpi data set starts very close to the peak viral load (±1 day). Thus, only one individual has pre-peak data, 17 individuals lose the peak viral load, and five individuals have at least one missing data point after peak viral load (viral down-slope). Finally, we studied the robustness of parameter estimates using an additional data set with only the peak and post-peak measurements as a proxy for data collection starting around symptom onset and, consequently, the most common data set obtained in clinical practice.

An important issue with fitting acute infection data is that typically, we do not know the time of infection and, thus, the times relative to infection when data was collected. To study this issue, we considered three scenarios for each artificial data set created above. First, we assumed that we do not know the infection time and estimate it (*t*_*inf*_ = est) from the data as we did above, but now we are using our reduced data sets. Here, the first measurement is assigned time 0, and we estimate infection before that. In the second case, we assume we know the time of infection, as estimated from the full data set, and thus, *t*_*inf*_ = 0 is the actual infection time. We fit the model to the various data sets and estimate model parameters (with *t*_*inf*_ = 0). Lastly, when data in the initial viral growth phase is missing, it is common practice to set the time of infection from the literature [22,23,48,120], e.g., by assuming the viral load peaks at the estimated median time of symptom onset, i.e., 5 dpi for SARS-CoV-2 [91–104]. Thus, for this third case, we only use the post-peak data set and assume the time of the observed peak viral load is day 5. While used in previous literature, this approach is crude as it ignores the individual variability in time to symptom onset. Here, we are testing the effect of fixing this time on the estimation of parameters and using a fixed value of 5 days biases against our objectives since we “know” from the full data set that the population estimate is 5.7 days.

**Fig 6:**
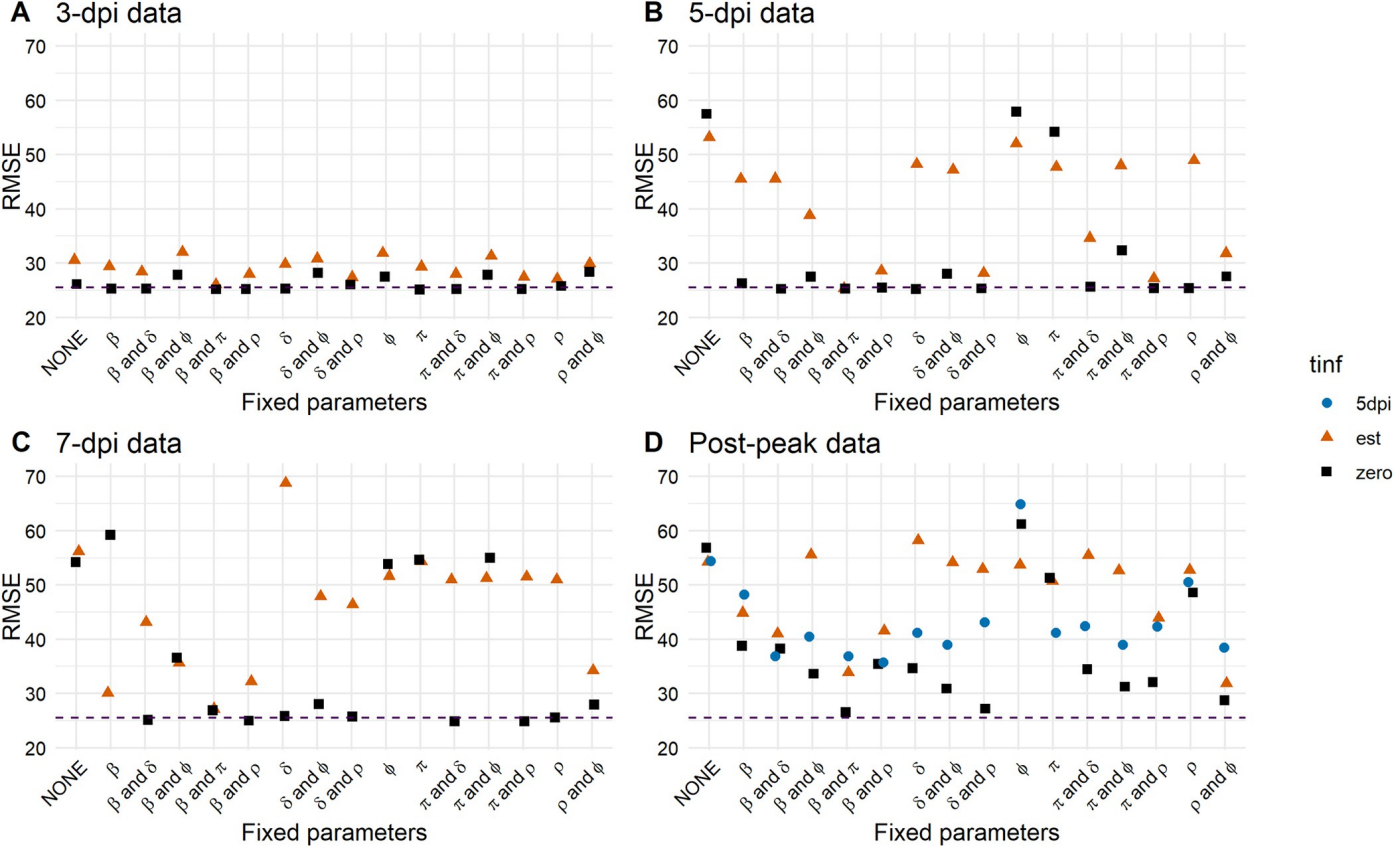
RMSEs for the RCM and three infection time cases. Infection times (t_inf_) are re-estimated (triangle), infection times are known and set to zero (square), or infection times are set to zero and the VL peaks 5 dpi (circle). RMSEs are shown for different subsets of the full data set: A) 3 dpi, B) 5 dpi, C) 7 dpi, and D) post-peak. For each case, all model parameters were re-estimated (NONE fixed on the x-axis), or the indicated model parameters were fixed to the values estimated from the full course of the infection data set and the remaining parameters estimated (see Table 1). The dashed line represents the RMSE calculated from the best model fit using the full course of infection (see S1 Data for RMSE values).

We fitted the model in turn to these different data sets and assumptions of *t*_*inf*_, and then evaluated how well the model with these newly estimated parameters describes the full data set, calculating the RMSE for the full data, as we did for the full model fit. Thus, we are calculating RMSE on the same full data set. The lower the RMSE, the better a model fitted to a subset of the data agrees with the full viral load course. We found, as expected, that the closer the data starts to the time of infection (*t*_*inf*_), i.e., the more data available pre-peak viral load, the lower the RMSE and the better the predictions align with those made with the full data set (Fig 6) [86]. Collecting data 3 dpi, 5 dpi, or 7 dpi, and knowing the time of infection (*t*_*inf*_ = 0) results in lower RMSEs than when estimating *t*_*inf*_ (Fig 6A–6D). However, the more viral load measurements pre-peak that are missing and without knowledge about the time of infection, the higher the RMSEs, leading to more uncertainty in the model prediction. Furthermore, for the post-peak viral load data set, if we assume that the viral load peaks 5 dpi and fix this (Fig 6D, blue dots), we obtain lower RMSEs compared to re-estimating infection times for each individual (Fig 6D, orange triangles), which in addition are usually poorly estimated. The TCLM yields similar results, but interestingly, in some cases, that model also yields slightly lower average RMSEs than the RCM (S1 Text). Note that the scenario assuming 5 days from infection to peak viral load was only fitted with the post-peak data.

Fixing one or more model parameters is a common practice to reduce uncertainty in data fitting [114]. Therefore, we were interested in how many model parameters, in addition to *c* and *k*, had to be fixed to describe the entire course of infection well with the different data sets. The parameters *c* and *k* are typically fixed as they refer to processes that take place on a timescale of minutes to hours for which data is unavailable [22,25,38,45,108,111,121]. Thus, we systematically fixed every single or possible pair of remaining model parameters to the population value estimated from the fit to the entire data set representing the entire course of infection (Table 1). Adding knowledge to the model fitting by fixing one or two additional parameters improved the RMSEs. Knowing *t*_*inf*_ yielded the lowest RMSE, followed by assuming the viral load peaks at 5 dpi, where both outperformed re-estimating *t*_*inf*_. By fixing the cell infection rate *β* and either the virus production rate *π* or the infected cell loss rate *δ* or the conversion rate of refractory cells into susceptible cells *ρ*, we generally observed the lowest RMSEs, which were close to those calculated from the whole course of infection data set (Fig 6A–6D). Especially *β* and *π* are crucial parameters of the initial growth phase. However, fixing only one of those model parameters (*β* or *π*) led to conflicting results. If infection times are not known and, thus, must be estimated, fixing only *β* yielded lower RMSEs than fixing only *π* when early data is missing (compare Fig 6A and 6B when early data is available with Fig 6C and 6D where only late data is available). However, if we assume the viral load peaks 5 dpi, fixing *π* yielded slightly lower RMSEs than fixing *β* (Fig 6D). Additionally, if infection times are re-estimated or if we assume the viral load peaks 5 dpi and fix *π*, the TCLM yielded lower RMSEs than the RCM (S1 Text).

### How robust are estimated infection times and other model parameters?

To evaluate the robustness of parameter estimates to missing data, we analyzed the parameters estimated with the modified data sets, with and without fixing model parameters (beyond *c* and *k*) and compared them to those estimated from the full course of infection. Note that we do not evaluate the biological accuracy of the estimated parameter values, which needs experimental and clinical validation, but rather studied the robustness or consistency of model parameters estimated from the data sets with varying numbers of data points pre-peak viral load. That is, we assume that the fit to the full data set is the ground truth, and thus the parameters estimated in that fit are “correct”.

**Fig 7:**
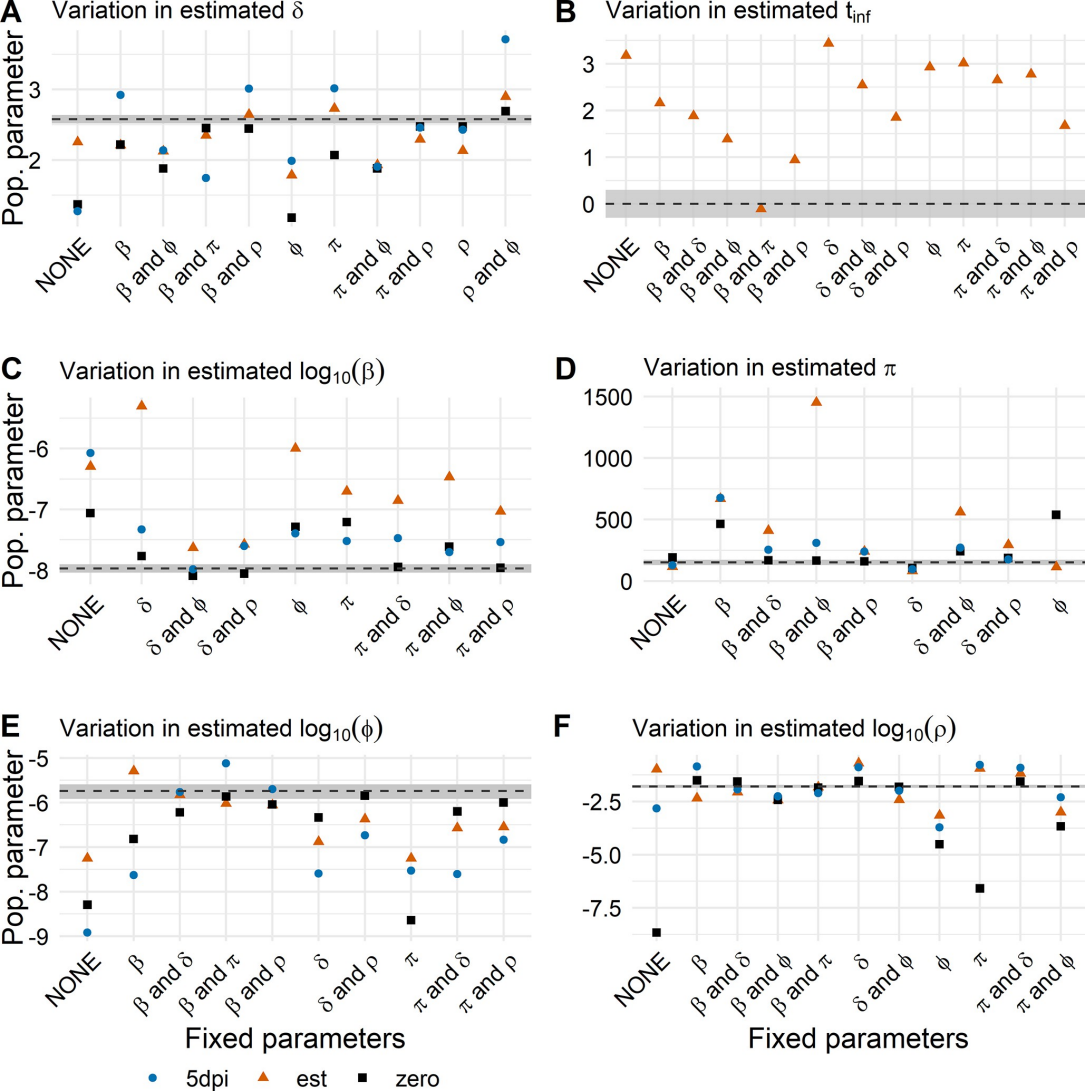
Population parameters estimates averaged over the four subsets of the full data set using the RCM. The dotted line represents the population parameter estimated from the “full course of infection” data set. The gray shaded area represents 95% confidence intervals of the population parameter estimated from the “full course of infection” data set (Population parameters estimated from each data subset individually can be found in Fig B in S2 Text and S1 Data).

The estimated *δ* was close to the value estimated from the full course of infection if *ρ* was fixed due to their correlation (Fig 7A and Fig B in S2 Text). Furthermore, only by fixing *β* and *π*, were we able to estimate the time of infection robustly and almost exactly (Fig 7B). Estimating *β* was most robust if we assume the viral load peaks 5 dpi and fix one or more model parameters (Fig 7C). Again, *π* was mostly over or underestimated when fixing one or two model parameters. However, not fixing any parameters led to the most robust estimate of *π* for all three studied cases (Fig 7D). For both innate immune response model parameters *φ* and *ρ*, fixing *β* and *π* or *β* and *δ* performed best for all subsets of the full data set (Fig 7E and 7F).

### Effect of choosing different combinations of model parameters

**Fig 8:**
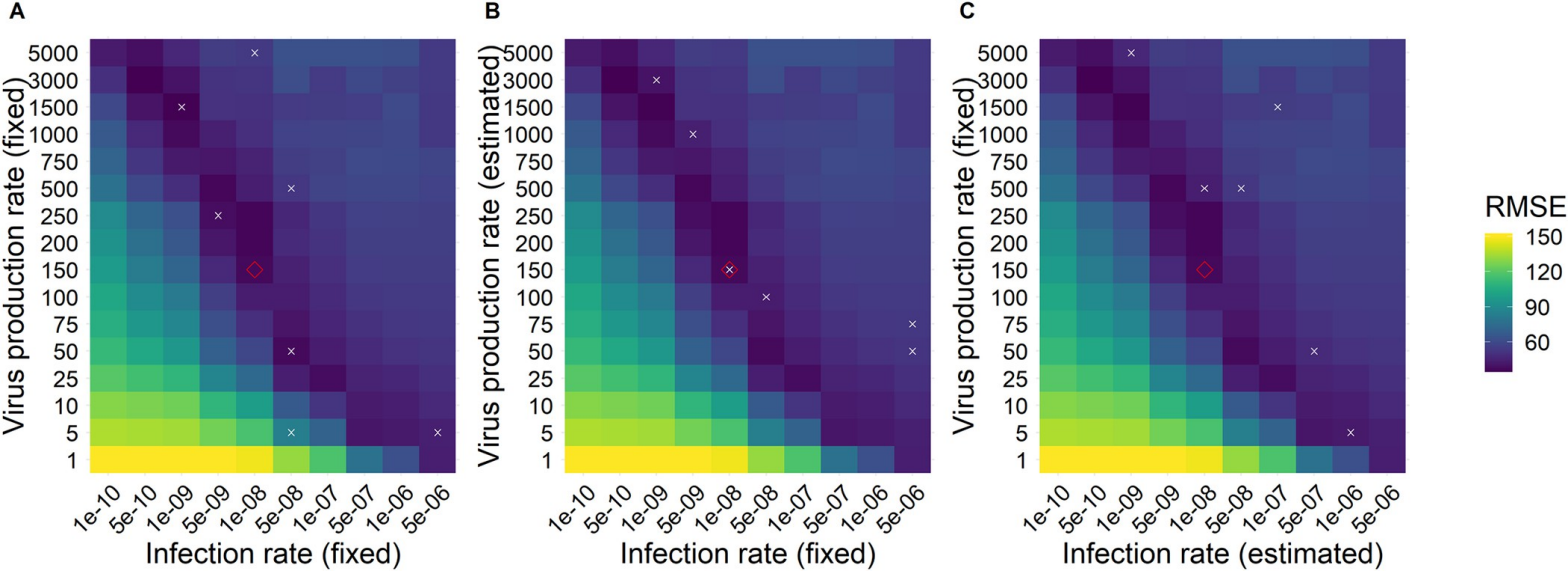
Heatmap RMSEs calculated with the RCM and different combinations of population parameters. A) RMSEs for different combinations of literature values. B) RMSEs for fixed infection rates from literature and estimated virus production rates. C) RMSEs for fixed virus production rates from literature and estimated infection rates. ◊ = the population parameters we estimated, x = values for β and/or π found in literature. Parameter values can be found in Table C in S2 Text. Note that the underlying heatmap is the same for A), B), and C), and it was calculated from fits to the post-peak viral load data set and assumed 5 days to reach peak viral load for each pair of β and π values indicated in the x and y-axes, respectively (see S1 Data for RMSE values).

Given that our results demonstrated the importance of knowing the cell infection rate (*β*) and the virus production rate (*π*), we were interested in the model performance when these are unknown but fixed at biologically plausible values. For that, we fixed *β* and *π* at various values and estimated the remaining model parameters, using the post-peak data set and assuming 5 days to reach peak viral load, as this is the most common study scenario. We then used the parameter estimates from these fits to simulate the full course of infection and calculate RMSE for the full course of infection. As shown in Fig 5, *β* and *π* correlate inversely, and because of this, we found that different combinations of *β* and *π* led to equally good fits, represented as dark blue tiles and, thus, low RMSEs (Fig 8). By visual inspection, we define fits with RMSEs within 20% of the best RMSE as equally good.

We include different values for *β* and *π* reported in the literature in these analyses and obtained using either the RCM or TCLM [1,12,21,24,44,120,122,123] (Table C in S2 Text, Fig 8). Compared to our estimate of *β* = 1×10^−8^ mL/RNA copies/day, the infection rate values found in the literature were mostly in the range between 1×10^−9^ to 5×10^−8^. These values and their corresponding virus production rates, which ranged between 50 and 1500 (our estimate *π* = 151 RNA copies/mL/day), led to equally good fits if *β*≥5×10^−8^ and *π*≥50 (Fig 8A). However, in the absence of pre-peak viral load data, we observed overall lower RMSEs, if fixing *β* and estimating *π* (Fig 8B, Table C in S2 Text) instead of fixing *π* and estimating *β* (Fig 8C, Table C in S2 Text). Thus, using infection rate values from the literature obtained using similar models and diverse data sets represents a good strategy to deal with missing data in the initial growth phase and missing information about infection times.

## Discussion

Estimating parameter values in viral dynamic models with missing data is challenging. Especially in acute infection, where individuals generally only become aware of being infected when symptoms develop. Thus, information about the time of infection and viral load measurements prior to symptom onset is often not available. The uncertainty of model predictions with missing data early post-infection was recently studied by Ciupe et al. for an acute infection (SARS-CoV-2) [86]. But they focused only on two scenarios of data availability, early and late, and on the estimate of virus infectivity, *β*. They found that the availability of early data post-infection and, thus, pre-peak viral load data improved the practical identifiability of *β*. However, they did not use a mixed effects approach [86] as in the current study, which improves identifiability properties [124]. Indeed, another recent study [89] evaluated the same issue of sparse initial data, but for chronic HIV-1, using a similar model and also using the same population mixed-effects approach we do. They found that information was gained across individuals, even when some had data missing, although uncertainty rose with fewer data points close to infection [89]. In our study, in addition to analyzing the robustness of estimated viral dynamic model parameters in the absence of variable amounts of data in the pre-peak growth phase, we also studied the effect of having extra information, such as the time of infection and/or knowing the values of some parameters. We found that viral infection and virus production rates are key parameters in determining the pre-peak viral growth rate, and with a lack of early data, the time to peak viral load was often underestimated. However, fixing the time of infection based on epidemiological studies represented a good alternative to estimating infection times and resulted in good model fits.

### Viral dynamics of the entire course of infection

The RCM describes the frequent viral load measurements of the 25 studied individuals well (Fig 3). Most estimated model parameters agreed with the estimates made in previous work, except for the transition rate of refractory cells back into susceptible cells, which we now estimate to be almost 3-fold higher [1]. Interestingly, we estimated that only 3% of the initial number of target cells were infected at the viral peak and 6% cumulatively from infection to peak viral load (Fig 4). In contrast, at the peak viral load, most cells were in a refractory state (81% of *T*_0_), only 12% of the initial number of target cells remained susceptible to infection, and the remainder of *T*_0_ were either infected cells or cells killed by the virus. This suggests that turning target cells into cells refractory to viral infection by establishing an antiviral state in uninfected cells may be a critical host defense mechanism early on in fighting a viral infection. And indeed, there are several reported mechanisms for a cell to accomplish this [105,107,125,126]. However, as far as we know, experimental measurements of the *in vivo* fraction of cells in an antiviral state during SARS-CoV-2 (or indeed any other) infection are not available and thus limit our ability to compare these predictions to data. Note that in the RCM, viral rebound occurs when refractory cells become susceptible again to infection. *In vivo*, the rebound is most likely prevented by adaptive immune responses that neutralize the free virus and kill infected cells. Due to a lack of data associated with the adaptive immune response, we did not model these mechanisms here.

### The effect of missing data in the pre-peak viral growth phase

With missing data in the pre-peak viral growth phase, infection times are underestimated by 2 to 3 days, resulting in very fast initial growth rates. Here, we show that estimation of the infection time can be improved by adding knowledge to the model. Cell infection and virus production rates are crucial parameters for describing the pre-peak growth phase. Fixing both model parameters to our population values led to infection time estimates similar to those estimated from the entire course of infection data set.

We were further interested in the robustness of other model parameter estimates to missing data. By fitting the RCM to subsets of the full data set, thus mimicking different data collection scenarios, we found that knowing the infection time led to the lowest RMSEs (Fig 6). However, a low RMSE did not guarantee the correct estimation of population parameters due to the correlations in the model parameters, such as the correlation between the cell infection rate (*β*) and the virus-production rate (*π*). Furthermore, the infection time is often unknown, but if one has knowledge of that time (e.g., from epidemiological studies) and uses it by fixing the infection time in the fitting, we obtain more robust results, which are better than estimating infection times. Estimated infection times were underestimated by up to 3 days when peak viral load data was missing while fixing *β* and *π* to values estimated from the full data set led to the most robust infection time estimates and closest to the value estimated with the full data set. Nevertheless, fixing a time to peak viral load of 5 days (for SARS-CoV-2 [91–104]) represented a good alternative to estimating infection times and led to estimates of the population parameters close to those estimated from the full course of infection data set. However, while we offer methods for estimating model parameters even without early viral load data, carefully gathered viral load measurements at different stages of infection data can enhance the precision and reliability of the model’s parameter estimates and predictions of infection times, incubation periods, and infectiousness.

Interestingly, whether infection times are known, estimated, or assumed, the loss rate of infected cells (δ) represented the most robust model parameter, with more consistent estimation, due to frequent viral load measurements after symptom onset and thus after peak viral load.

### Estimating *β* and *π*: What if *β* and *π* are unknown?

The cell infection rate *β* and virus production rate *π* are crucial parameters of the pre-peak viral growth phase. Fixing both model parameters may lead to robust predictions of infection times (Fig 7). However, both model parameters are often unknown and challenging to measure experimentally.

If infection times are unknown, assuming 5 days from the time of infection to peak viral load led to the most robust estimates of *β*. Nevertheless, estimates of *π* showed more variability, possibly due to lower sensitivity. It has been previously shown that the initial target cell population *T*(*t*_*inf*_) also correlates with the virus production rate *π* and only their product [*T*(*t*_*inf*_)⋅*π*] is identifiable [87,90,109,127].

Encouragingly, using estimates for the cell infection and virus production rates from other modeling studies that used similar RCM and TCLM models [1,12,21,24,44,120,122,123] led to equally good fits of the data, for example, *β* = 10^−8^ mL/RNA copies/day and *π* = 150 to 200 RNA copies/mL/ day or *β* = 10^−9^ mL/RNA copies/day and *π* = 1000 to 1500 RNA copies/mL/day (Fig 8 and Table C in S2 Text). Consequently, when there is missing data in the pre-peak growth phase, taking cell infection and virus production rates from other modeling studies in the literature that used similar models may allow robust predictions of the viral load dynamics during the growth phase.

### Limitations and outlook

Our analysis was based on models of infection similar to those used for a variety of viruses, including West Nile virus [27], Zika [35], respiratory syncytial virus [36], influenza [25,38–41], and SARS-CoV-2 [1,12,22,24,43], as well as primary infection in HIV [89] and HBV [66], among others. Still, we only analyzed in detail two models, the TCLM and the RCM, and not all our conclusions may extend to variations of these models. We based our study on a specific data set for SARS-CoV-2 infection due to its availability and richness. However, we claim that our results are a property of the models and not of the specific data set, and we could have just used synthetic data (generated by model simulation) to obtain the same conclusions (as in [86]). A more important limitation is that we analyzed the effect of extra information, which may not be available for some infections, especially emergent infections. However, if nothing or very little is known, modeling is inherently difficult, and we show that at least “guessing” the time to the viral peak, and fixing it in the fitting procedure, will inject some robustness into the results. Another issue is that we specifically did not include an adaptive immune response in our models, which for example precluded us from using data from vaccinated individuals. The reason is that the immune response is often quite variable among individuals and may result in an additional level of uncertainty [12].

Overall, our results may be useful in guiding data collection and using that data to best estimate viral dynamic parameters such as the death rate of infected cells, which can inform about viral pathogenesis. Moreover, if a novel virus emerges in the future, we emphasize that one should strive to obtain the most informative data sets, i.e., frequent measurements throughout the course of infection, so that one can have the most confidence in any inference about the infection kinetics and the infectious period of an individual. Therefore, in the event of a novel virus emergence, prioritizing data collection with frequent viral load measurements beginning as early as possible and continuing throughout the course of infection can improve model predictions and, hence, our understanding of the virus’ behavior to respond effectively to the evolving situation.

In summary, the current study provides new insights into viral dynamic modeling in the absence of frequent viral load measurements, particularly in the early stages of infection. We evaluated the robustness of estimated model parameters to missing data and found that cell infection and virus production rates are key parameters of the viral growth phase that are difficult to estimate without early data. Furthermore, missing data before the viral load peaks leads to underestimates of the time to peak viral load and to inconsistent estimated model parameters. However, fixing infection times from epidemiological studies and/or model parameters that are important in determining the viral growth rate (*β* and *π*) represented a good alternative and led to robust model fits and model parameter estimates.

## Supporting information

S1 Text The target cell limited model. (DOCX)

S2 Text Supporting information. (DOCX)

S1 Data Experimental measurements and model predictions. (XLSX)
