# Supplementary material for: How robust are estimates of key parameters in standard viral dynamic models?
Source: PLoS Comput Biol. 2024 Apr 16;20(4):e1011437. doi: 10.1371/journal.pcbi.1011437 (PMC11051641; doi:10.1371/journal.pcbi.1011437)
Supplement: S1 Text — (DOCX) [file pcbi.1011437.s001.docx]

S1 Text: The target cell limited model.

Besides fitting the refractory cell model (RCM) to frequent viral load measurements of 25 selected individuals infected with SARS-CoV-2 (see Main text), we also studied the simplified version of the RCM, the target cell limited model (TCLM), which lacks refractory cells.

## Model description

The TCLM describes the dynamics of target cells, i.e., cells susceptible to infection, $T$, infected cells in the eclipse phase that are not yet virus-producing, $E$, virus-producing infected cells, $I$, and virus, $V$ (Fig A). The dynamics of the four populations is given by the following system of ordinary differential equations (ODEs):

$$\begin{aligned} \frac{dT}{dt}= -\beta TV,\#\left( S1 \right) \end{aligned}$$

$$\frac{dE}{dt}=\beta TV-kE,$$

$$\frac{dI}{dt}=kE-\delta I,$$

$$\frac{dV}{dt}=\pi I-cV.$$

Target cells become infected by virus with rate constant $\beta$. After an eclipse phase with duration $1/k$, infected cells produce virus with rate constant $\pi$ and die with rate constant $\delta$. Virus is cleared with rate constant $c$.

## Model performance of the TCLM

The TCLM fits the viral load data of the 25 selected individuals well (Fig B). However, the RCM fit the data better than the TCLM, yielding overall lower RMSE (root-mean-square error): RMSE_RCM_ = 24.3 compared to RMSE_TCLM_ = 26.6, respectively. Additionally, the corrected Bayesian Information Criterion (BICc), which penalizes the number of model parameters, was lower for the RCM (BICc = 980) with 6 parameters compared to the TCLM (BICc =986) with 4 parameters and thus preferring the RCM.

In the original data set, where time 0 represented the measured peak viral load, we estimated infection a median of -7.6 days pre-peak with the TCLM (individually ranging between -10.8 to -6.5 days) for the whole population (Tables A and B). However, we estimated the peak viral load was reached an average of 6.5 days after infection suggesting the highest measured viral load is not necessarily the true peak viral load (Fig C). Furthermore, with the TCLM, we estimated the post peak clearance time to be 8.4 days to undetectable viral load. The estimated infection duration was around 15 days.

Combined with the eclipse phase duration, $1/k$, the average lifespan of infected cells is $\frac{1}{k} +\frac{1}{\delta}=1$ day in the TCLM compared to the RCM with an average lifespan of infected cells of 0.64 days or 15 hours.

Interestingly, with the TCLM we estimated a within-host $R_{0}$ of 8, which is consistent with but slightly higher than the *R*_0_=5 with the RCM. Compared to the TCLM, the RCM estimated faster rates of virus production ($\pi^{TCLM}=3$ versus $\pi^{RCM}=151$ RNA copies/mL/day) and death of infected cells ($\delta^{TCLM}=1.28$ versus $\delta^{RCM}=2.58$ /day), but a slower cell infection rate ($\beta^{TCLM}=4.27\times{10}^{-7}$versus $\beta^{RCM}=1.07\times{10}^{-8}$ mL/RNA copies/day), resulting in an on average one day shorter time to peak (6.5 days with TCLM versus 5.7 days with RCM) and thus lower within-host transmission ($R_{0}^{TCLM}=8$ versus $R_{0}^{RCM}=5$) (Table 1).

The infected cell population peaks around 6 dpi with on average 58% of cells infected and a depleted population of target cells with less than 1% susceptible cells left (Fig C).

Similar to the RCM, we found several model parameters significantly correlated in the TCLM (Fig D). For example, the cell infection rate constant ($\beta$) and the virus production rate ($\pi$) are negatively correlated and $\pi$ is further positively correlated with the loss rate of infected cells ($\delta$).

## Model prediction with missing data

With missing data in the initial viral growth phase and for all three infection time scenarios – time to peak viral load known, re-estimated or assuming it takes 5 days to the peak viral load – the TCLM yields lower average RMSEs (again calculated for the full data set) than the RCM. Additionally, if infection times are unknown or if we assume the viral load peaks 5 dpi and fix either $\beta$ or $\pi$, the TCLM yielded overall lower RMSEs than the RCM.

Estimates of $\delta,$ the loss rate of infected cells, from our four data collection scenarios (3, 5, 7, and post-peak) were the most robust (Figs E and F). With or without fixing model parameters, estimates for $\delta$ are mostly around its value estimated from the whole course of infection (1.28 ± 0.128 /day). As the rate of viral load decay post-peak is heavily influenced by $\delta$ this result is not surprising. Interestingly, estimated infection times were only close to those estimated from the entire course of infection if $\beta$ is fixed (Fig G). Estimating $\beta$ correctly was only possible with the assumption that the viral load peaks 5 dpi or in most cases if infection times are known. The estimates for $\pi$ were typically overestimated by one or two orders of magnitude the more data was missing pre-peak viral load.


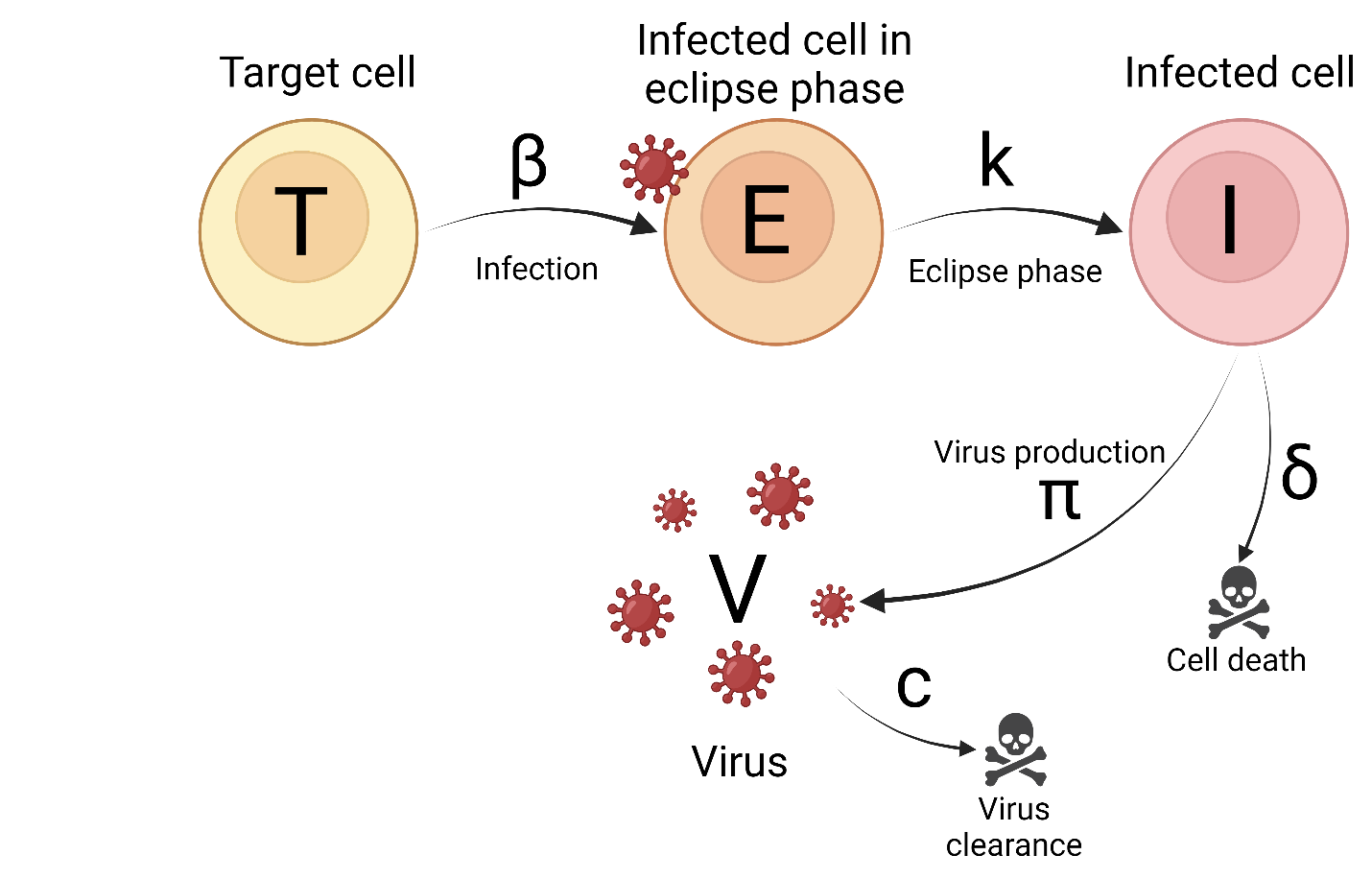


**Fig A: Schematic illustration of the target cell limited model extended by an eclipse phase.** A susceptible target cell, $T$, is infected by virus, $V$, with the infection rate constant $\beta$. Infected cells in the eclipse phase, $E$, become actively virus producing cells, $I$, with the transition rate constant $k$. $I$ produce virus with production rate constant $\pi$ or die with degradation rate $\delta$. Virus is cleared with clearance rate $c$. [Created with BioRender.com]


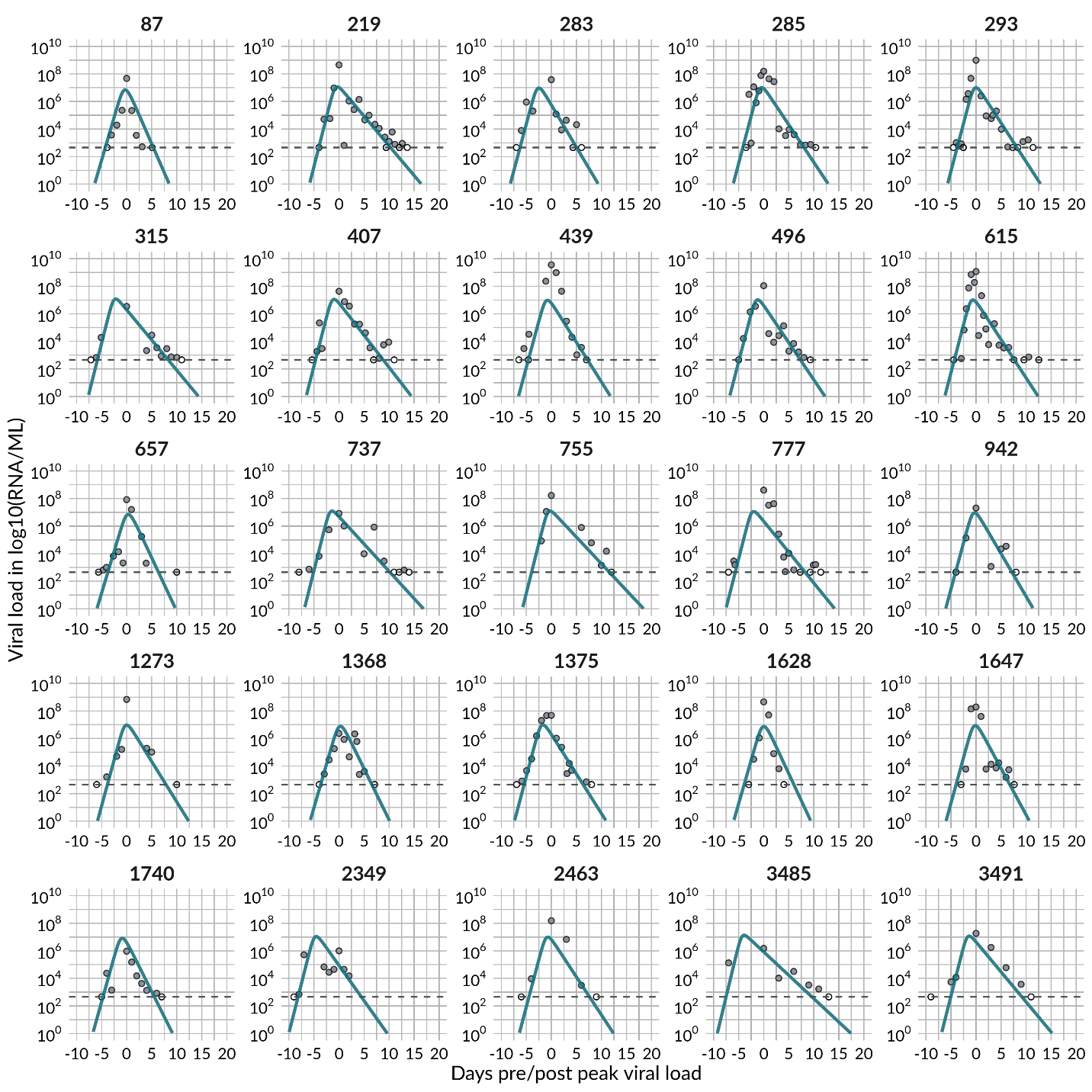


**Fig B: The best TCLM fit to viral load measurements of 25 selected individuals.** Filled circles are measurement points, and non-filled circles are censored and below the detection limit (dotted grey line).

**Table A: Population parameters in the viral dynamic models and their estimated values.**

| **Parameter** | **Description** | **TCLM**  **Population estimate**  **[95% CI]** | **Unit** | **References** |
| --- | --- | --- | --- | --- |
| $T\left( t_{inf} \right)$ | Initial target cell population | $\boldsymbol{8\times}\boldsymbol{10}^{\boldsymbol{7}}$ | cells | [1–3] |
| $E\left( t_{inf} \right)$ | Initial number of infected cells in the eclipse phase population | **1** | cells | [1] and Table A in S2 Text |
| $t_{inf}$ | Infection time | -7.6  [-8.2, -7.0] | days |  |
| $\beta$ | Cell infection rate | $4.27\times{10}^{-7}$  [3.63$\times{10}^{-7}$ , 5.13 $\times{10}^{-7}$ ] | mL/RNA copies/day |  |
| $k$ | Eclipse phase duration | **4** | 1/day | [1,2,4] |
| $\pi$ | Virus production rate | 3.07  [2.76, 3.41] | RNA copies/mL/day |  |
| $\delta$ | Death rate of infected cells | 1.28  [1.20, 1.35] | 1/day |  |
| $c$ | Virus clearance rate | **10** | 1/day | [1,4] |
| $R_{0}$ | Basic reproductive number | 8.2 |  |  |
| RMSE | Root mean squared error  Sum over all individuals:  Averaged individual: | 26.6  1.06 |  |  |
| -LL  BICc | negative log likelihood:  corrected Bayesian Information Criterion: | 944.44  986.05 |  |  |

*Parameter values in bold were fixed.*

**Table B: Individual parameters in the TCLM and their estimated values.**

| ***ID*** | $\boldsymbol{t}_{\boldsymbol{inf}}$ | $\boldsymbol{\delta}$ | $\boldsymbol{\pi}$ | $\boldsymbol{10}^{\boldsymbol{\beta}}$ | ***time of first measured VL below LOD*** |
| --- | --- | --- | --- | --- | --- |
| 87 | -7.3 | 2.05 | 3.09 | -6.36 | -3.9 |
| 219 | -6.7 | 0.99 | 3.06 | -6.37 | -4.0 |
| 283 | -9.1 | 1.42 | 3.09 | -6.36 | -7.0 |
| 285 | -6.9 | 1.26 | 3.06 | -6.37 | -3.5 |
| 293 | -6.5 | 1.3 | 3.07 | -6.37 | -4.5 |
| 315 | -8.5 | 1.01 | 3.08 | -6.37 | -7.1 |
| 407 | -7.4 | 1.08 | 3.06 | -6.37 | -5.4 |
| 439 | -7.4 | 1.33 | 3.05 | -6.38 | -6.5 |
| 496 | -7.7 | 1.25 | 3.08 | -6.36 | -5.0 |
| 615 | -7.1 | 1.29 | 3.08 | -6.36 | -4.5 |
| 657 | -6.9 | 1.89 | 3.03 | -6.38 | -5.6 |
| 737 | -7.7 | 0.91 | 3.06 | -6.37 | -8.0 |
| 755 | -6.6 | 0.89 | 3.05 | -6.37 | missing |
| 777 | -8.4 | 1.03 | 3.07 | -6.37 | -7.0 |
| 942 | -6.9 | 1.44 | 3.07 | -6.37 | -4.0 |
| 1273 | -6.7 | 1.36 | 3.05 | -6.38 | -6.0 |
| 1368 | -6.7 | 1.78 | 3.05 | -6.37 | -4.0 |
| 1375 | -8.2 | 1.33 | 3.08 | -6.37 | -6.9 |
| 1628 | -6.9 | 1.88 | 3.08 | -6.36 | -3.0 |
| 1647 | -6.9 | 1.58 | 3.07 | -6.37 | -3.0 |
| 1740 | -7.7 | 1.72 | 3.08 | -6.36 | -5.0 |
| 2349 | -10.8 | 1.18 | 3.11 | -6.36 | -9.0 |
| 2463 | -7.3 | 1.28 | 3.06 | -6.37 | -6.0 |
| 3485 | -10.1 | 0.78 | 3.09 | -6.36 | -11.0 |
| 3491 | -7.7 | 1 | 3.06 | -6.37 | -9.0 |


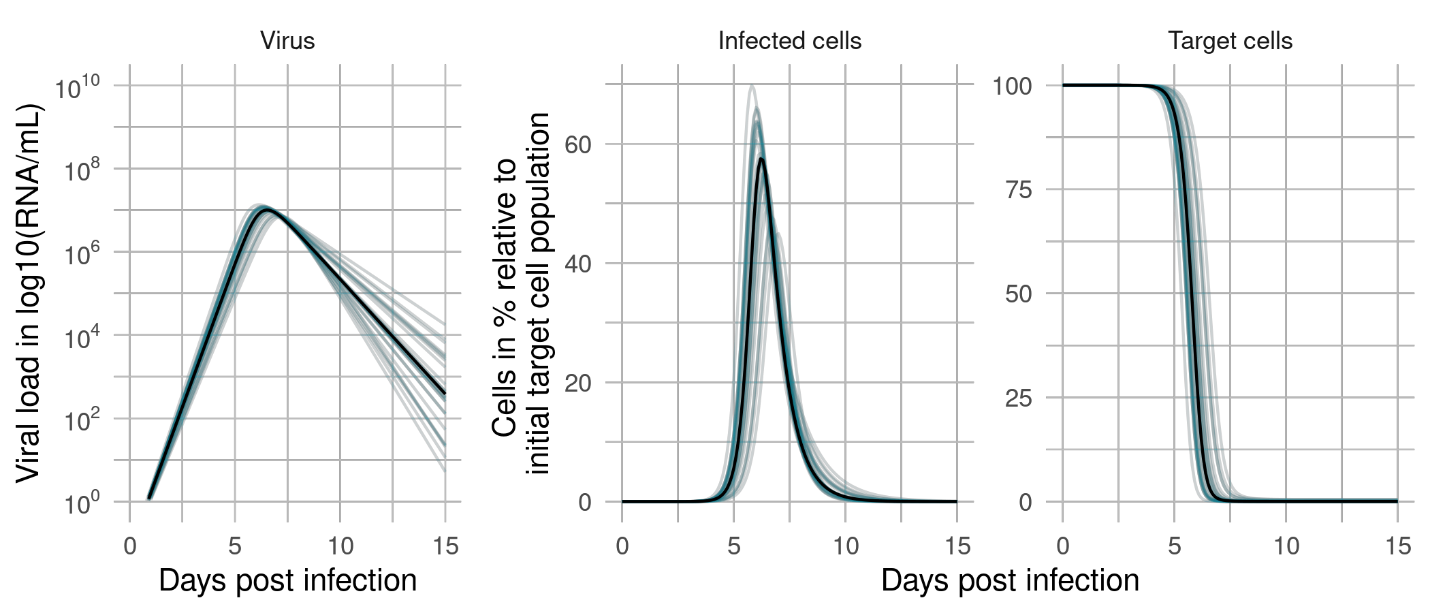


**Fig C: Virus and cell dynamics.** Predicted dynamics of infected cells, refractory cells, and target cells using the population parameter (black line) and individual parameters (colored lines) throughout the course of infection predicted by the target cell limited model using the best-fit population parameter estimates.


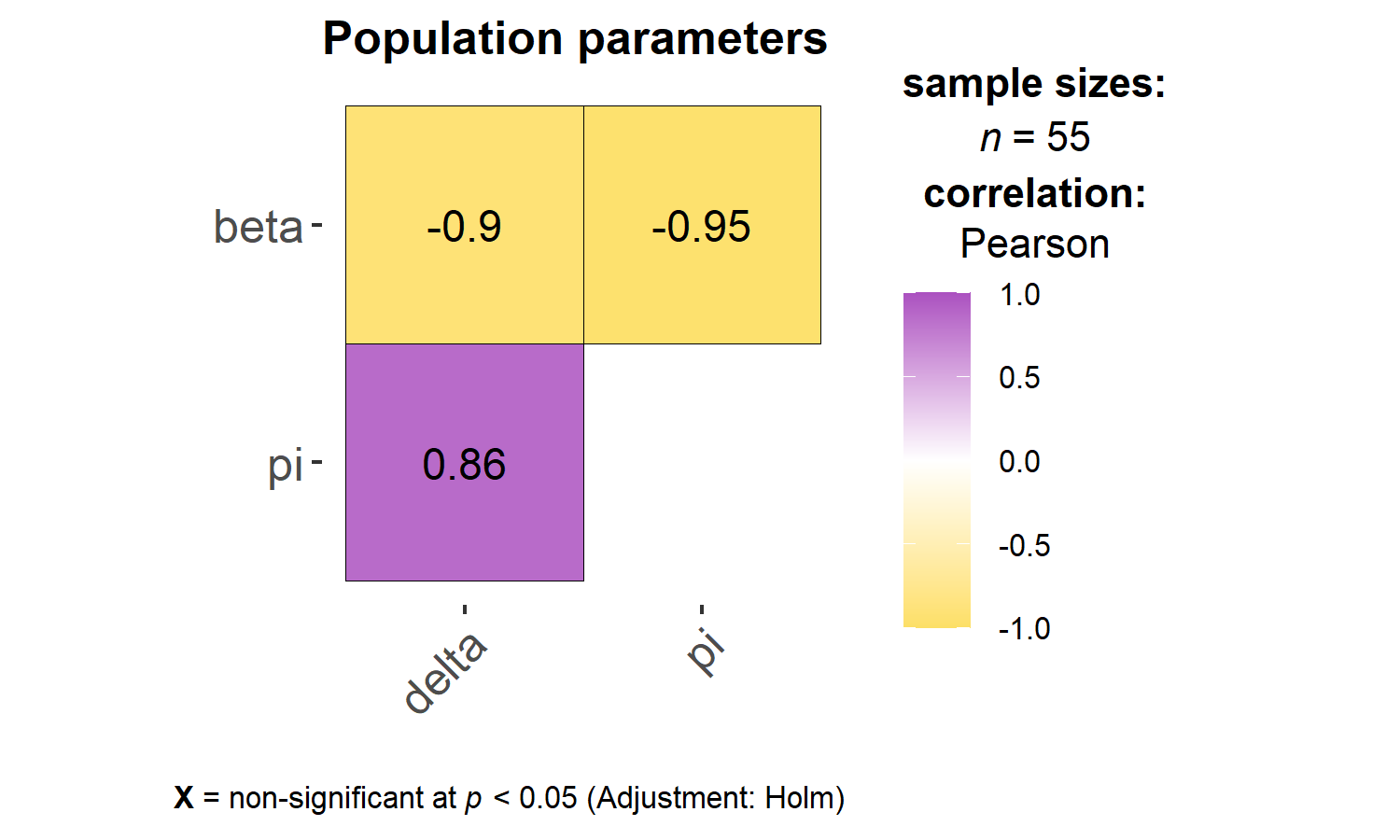


**Fig D: Correlation of population parameters in the target cell limited model.** The sample size gives the number of fits that fit the model equally well in the range of min(-LL)+2. Correlations that are crossed are non-significant (p-value > 0.05). The plot has been generated with ggstatsplot [doi:10.21105/joss.03167]


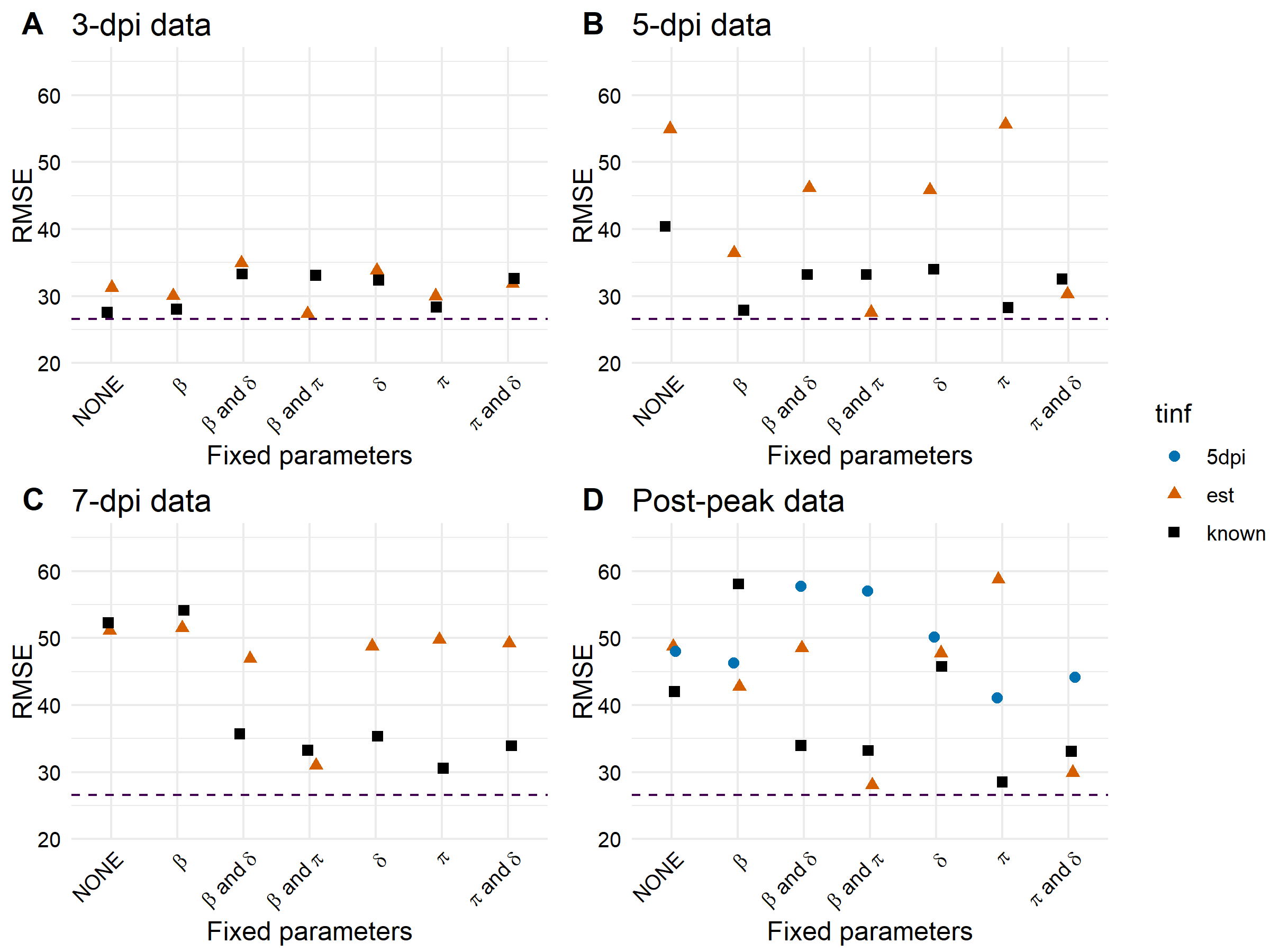


***Fig E: RMSEs for the TCLM and three infection time cases****. Infection times (t_inf_) are re-estimated (triangle), infection times (t_inf_) are known and set to zero (square), or infection times are set to zero and the VL peaks 5 dpi (circle).* *RMSEs are shown for different subsets of the full data set: A) 3 dpi, B) 5 dpi, C) 7 dpi, and D) post-peak. For each case, all model parameters were re-estimated (NONE on the x-axis), or model parameters were fixed to the values estimated from the full course of the infection data set (see Table A).* *The dashed line represents the RMSE calculated from the best model fit using the full course of infection.*


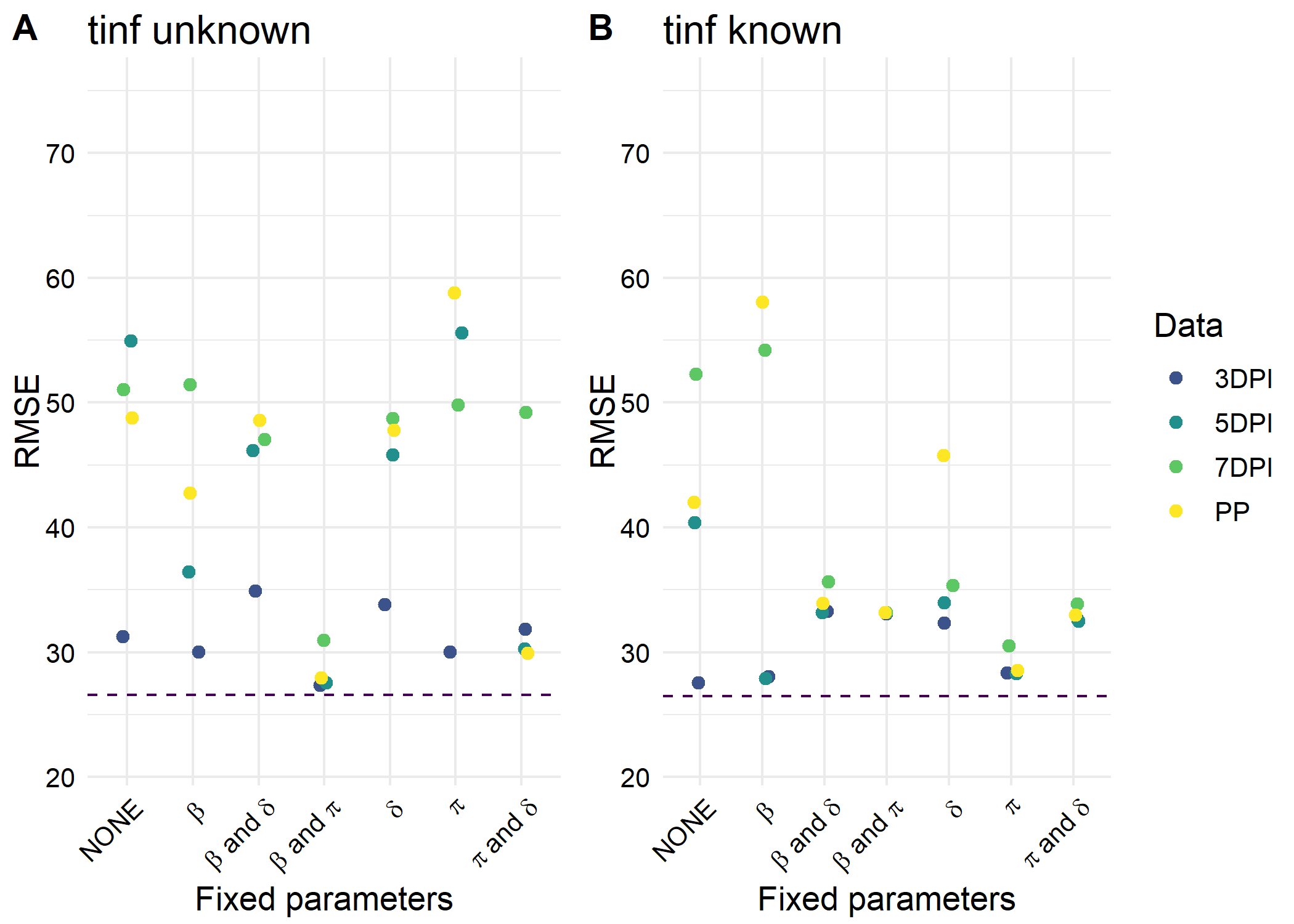


***Fig F:*** ***RMSEs for TCLM and different data subsets.*** *RMSEs for TCLM and the different data subsets and two infection time circumstances A) infection times (*$t_{inf}$*) are re-estimated or B) infection times (*$t_{inf}$*) are known and set to zero. For each data set, all model parameters are re-estimated (NONE on x-axis) or model parameters were fixed to the values estimated from the full course of infection data set (see Table A).*


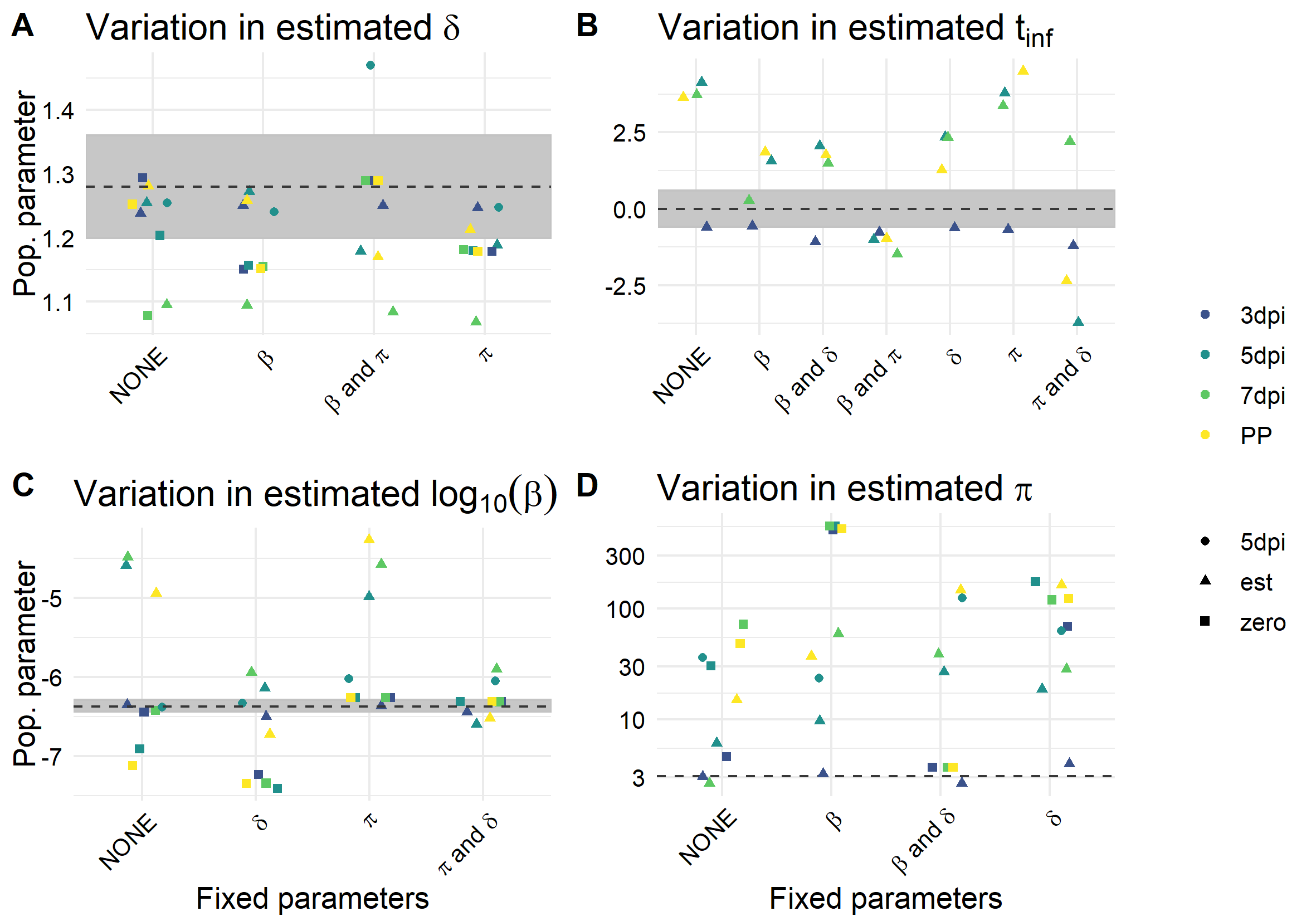


***Fig G: Estimated population parameters of the TCLM and data subsets starting 3, 5, 7, dpi, or post-peak.*** *The dotted line represents the population parameter estimated from the “full course of infection” data set (Y axis are estimated population values). The gray shaded area represents 95% confidence intervals of the population parameter estimated from the “full course of infection” data set. Note that the color decodes different data sets used for parameter estimation, while the shape decodes different infection times assumptions.*

## References

1. Ke R, Zitzmann C, Ho DD, Ribeiro RM, Perelson AS. In vivo kinetics of SARS-CoV-2 infection and its relationship with a person’s infectiousness. Proc Natl Acad Sci U S A. 2021;118. doi:10.1073/PNAS.2111477118/-/DCSUPPLEMENTAL

2. Baccam P, Beauchemin C, Macken CA, Hayden FG, Perelson AS. Kinetics of influenza A virus infection in humans. J Virol. 2006;80: 7590–9. doi:10.1128/JVI.01623-05

3. Hou YJ, Okuda K, Edwards CE, Martinez DR, Asakura T, Dinnon KH, et al. SARS-CoV-2 reverse genetics reveals a variable infection gradient in the respiratory tract. Cell. 2020;182: 429. doi:10.1016/J.CELL.2020.05.042

4. Gonçalves A, Bertrand J, Ke R, Comets E, Lamballerie X, Malvy D, et al. Timing of antiviral treatment initiation is critical to reduce SARS‐CoV‐2 viral load. CPT Pharmacometrics Syst Pharmacol. 2020;9: 509–514. doi:10.1002/psp4.12543
