# Supplementary material for: How robust are estimates of key parameters in standard viral dynamic models?
Source: PLoS Comput Biol. 2024 Apr 16;20(4):e1011437. doi: 10.1371/journal.pcbi.1011437 (PMC11051641; doi:10.1371/journal.pcbi.1011437)
Supplement: S2 Text — (DOCX) [file pcbi.1011437.s002.docx]

S2 Text: Supporting information.

Contents

[Sensitivity to the number of infected cells 1](#_Toc157074364)

[Best model fit with the peak viral load at t = 0 2](#_Toc157074365)

[Individual parameters in the TCM 3](#_Toc157074366)

[Estimated population parameters of the RCM and different data subsets 4](#_Toc157074367)

[Individual data selection process and Monolix model 5](#_Toc157074368)

[Data subsets of the full data set 6](#_Toc157074369)

[Profile likelihood estimation 10](#_Toc157074370)

[Estimated beta and pi values from the literature 17](#_Toc157074371)

[References 18](#_Toc157074372)

# Sensitivity to the number of infected cells

**Table A: Sensitivity of the fit to the initial number of infected cells** $\boldsymbol{E(}\boldsymbol{t}_{\boldsymbol{inf}}\boldsymbol{)}$**.** Highlighted in orange is the best fit. [-LL = negative log likelihood, BICc = corrected Bayesian Information Criterion, TCLM = Target cell limited model, RCM = Refractory cell model]

| $E(t_{inf})$ | TCLM | | RCM | |
| --- | --- | --- | --- | --- |
|  | -LL | BICc | -LL | BICc |
| 1 | 944.4 | 986.1 | 921.3 | 980.8 |
| 5 | 944.5 | 986.1 | 922.6 | 982.1 |
| 10 | 944.4 | 986.1 | 923 | 982.1 |
| 50 | 950.3 | 991.9 | 929.3 | 988.8 |
| 100 | 945.6 | 987.2 | 934.1 | 993.6 |

# Best model fit with the peak viral load at t = 0


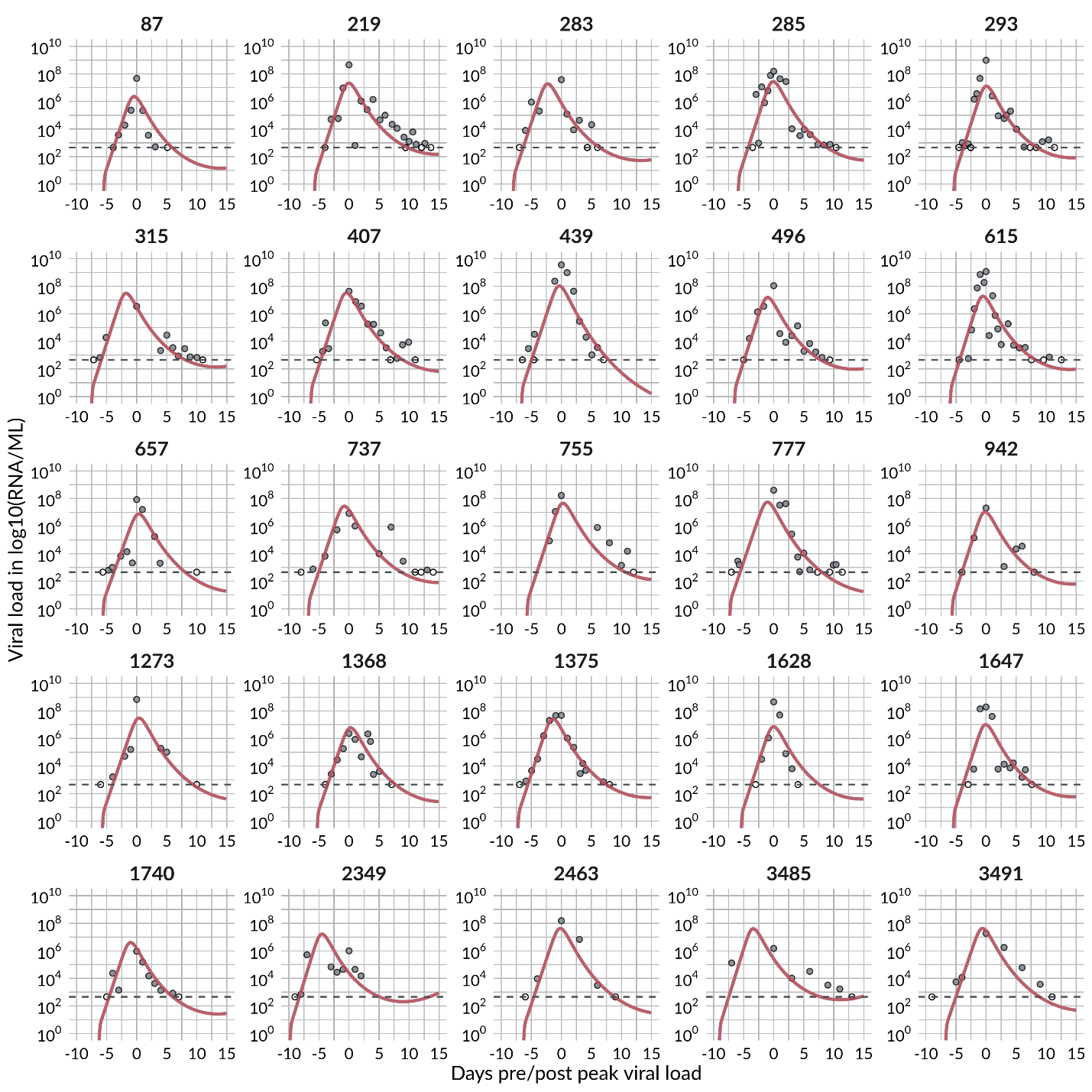


**Fig A:** **Best model fit with peak viral load at t = 0.** The best model fit to viral load measurements of 25 selected individuals with $t=0$ corresponds to the measured peak viral load. Filled circles are measurement points, and non-filled circles are censored and below the detection limit (dotted grey line).

# Individual parameters in the TCM

**Table B: Individual parameters in the RCM and their estimated values.**

| IDs | $t_{inf}$ | $\log_{10} (\varphi)$ | $\log_{10} (\rho)$ | $\delta$ | $\pi$ | $\log_{10} (\beta)$ | *time of first measured viral load below LOD* | *time of first measured viral load above LOD* |
| --- | --- | --- | --- | --- | --- | --- | --- | --- |
| 87 | -5.5 | -4.78 | -1.79 | 2.66 | 150.6 | -7.96 | -3.9 | -3.1 |
| 219 | -5.7 | -5.76 | -1.79 | 2.46 | 150.5 | -7.97 | -4.0 | -3.0 |
| 283 | -8 | -5.72 | -1.79 | 2.65 | 151.4 | -7.97 | -7.0 | -6.0 |
| 285 | -5.9 | -5.89 | -1.79 | 2.64 | 151.7 | -7.96 | -3.5 | -2.9 |
| 293 | -5.4 | -5.5 | -1.79 | 2.6 | 152.9 | -7.95 | -4.5 | -4.0 |
| 315 | -7.5 | -5.91 | -1.79 | 2.48 | 152.2 | -7.97 | -7.1 | -6.1 |
| 407 | -6.4 | -5.98 | -1.79 | 2.56 | 150.7 | -7.97 | -5.4 | -4.4 |
| 439 | -6.6 | -6.63 | -1.79 | 2.68 | 151.9 | -7.96 | -6.5 | -5.5 |
| 496 | -6.5 | -5.59 | -1.79 | 2.56 | 152.1 | -7.96 | -5.0 | -5.0 |
| 615 | -6 | -5.66 | -1.79 | 2.62 | 153.9 | -7.95 | -4.5 | -2.9 |
| 657 | -5.6 | -5.4 | -1.79 | 2.71 | 146.6 | -8.00 | -5.6 | -4.7 |
| 737 | -6.8 | -5.92 | -1.78 | 2.53 | 149.5 | -7.98 | -8.0 | -6.0 |
| 755 | -5.7 | -6.12 | -1.78 | 2.4 | 150.5 | -7.98 | missing | -1.0 |
| 777 | -7.3 | -6.28 | -1.79 | 2.61 | 150 | -7.98 | -7.0 | -2.0 |
| 942 | -5.7 | -5.45 | -1.79 | 2.6 | 151 | -7.97 | -4.0 | -2.0 |
| 1273 | -5.7 | -5.98 | -1.79 | 2.64 | 149.2 | -7.98 | -6.0 | -4.0 |
| 1368 | -5.3 | -5.23 | -1.79 | 2.65 | 148.7 | -7.98 | -4.0 | -3.0 |
| 1375 | -7.2 | -5.87 | -1.79 | 2.64 | 151.3 | -7.97 | -6.9 | -5.9 |
| 1628 | -5.4 | -5.27 | -1.79 | 2.64 | 151.2 | -7.96 | -3.0 | -2.0 |
| 1647 | -5.4 | -5.41 | -1.79 | 2.63 | 152.2 | -7.96 | -3.0 | -2.1 |
| 1740 | -6.3 | -5.02 | -1.79 | 2.63 | 150.5 | -7.97 | -5.0 | -4.0 |
| 2349 | -9.8 | -5.58 | -1.79 | 2.47 | 154.1 | -7.95 | -9.0 | -8.0 |
| 2463 | -6.3 | -6.12 | -1.79 | 2.63 | 150.6 | -7.97 | -6.0 | -4.0 |
| 3485 | -9 | -6 | -1.78 | 2.38 | 153.3 | -7.96 | -11.0 | -7.0 |
| 3491 | -6.7 | -6.12 | -1.79 | 2.53 | 149.4 | -7.98 | -9.0 | -5.0 |
| Mean |  |  |  |  |  |  | -5.8 | -4.1 |

# Estimated population parameters of the RCM and different data subsets


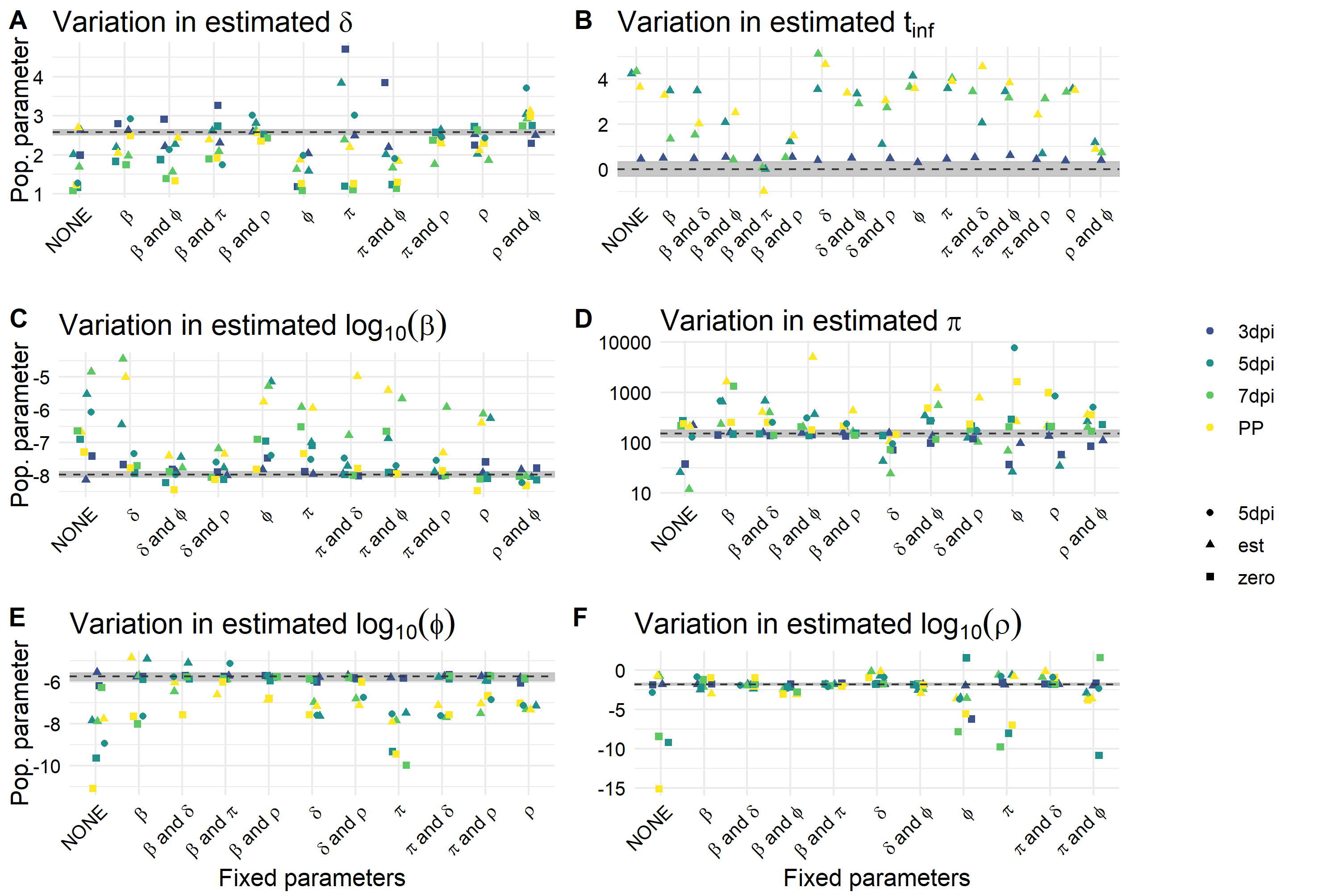


***Fig B: Estimated population parameters of the RCM and data collected 3, 5, 7 dpi, or post-peak.*** *The dotted line represents the population parameter estimated from the full course of infection data set (Y axis are estimated population values).*

# Individual data selection process and Monolix model

***Individual data selection process***

One hundred and ninety-one unvaccinated individuals were available from a total of 228 individuals, using a combination of the data sets in [1,2]. Forty-nine of them had a minimum of 4 measurements above the LOD, with at least one data point pre peak and one post peak viral load above the limit of detection, and no measurement above the LOD more than 14 days pre- or post-peak viral load. For our main analysis, we selected randomly 25 of these individuals (see main text).

***Monolix model***

We estimated the following parameters in Monolix:

input = {T0, E0, tt0, logphi, logrho, delta, p, c, logbeta}

And we used the following equations:

ddt_T = – (10^logbeta) *V*T – (10^logphi)*T*I + (10^logrho)*R

ddt_R = (10^logphi)*T*I – (10^logrho)*R

ddt_E = (10^logbeta)*V*T – k*E

ddt_I = k*E – delta*I

ddt_V = p*I – c*V

with initial conditions

t0 = – tt0

T_0 = T0

R_0 = 0

E_0 = E0

I_0 = 0

V_0 = 0

and fixed model parameters

k = 4

T0 = 8e+07

E0 = 1

c = 10

With this function, we fitted the log10(V) to the logarithm of the observed viral load (VL).

# Data subsets of the full data set

***Data subset where data collection starts 3 days post infection***
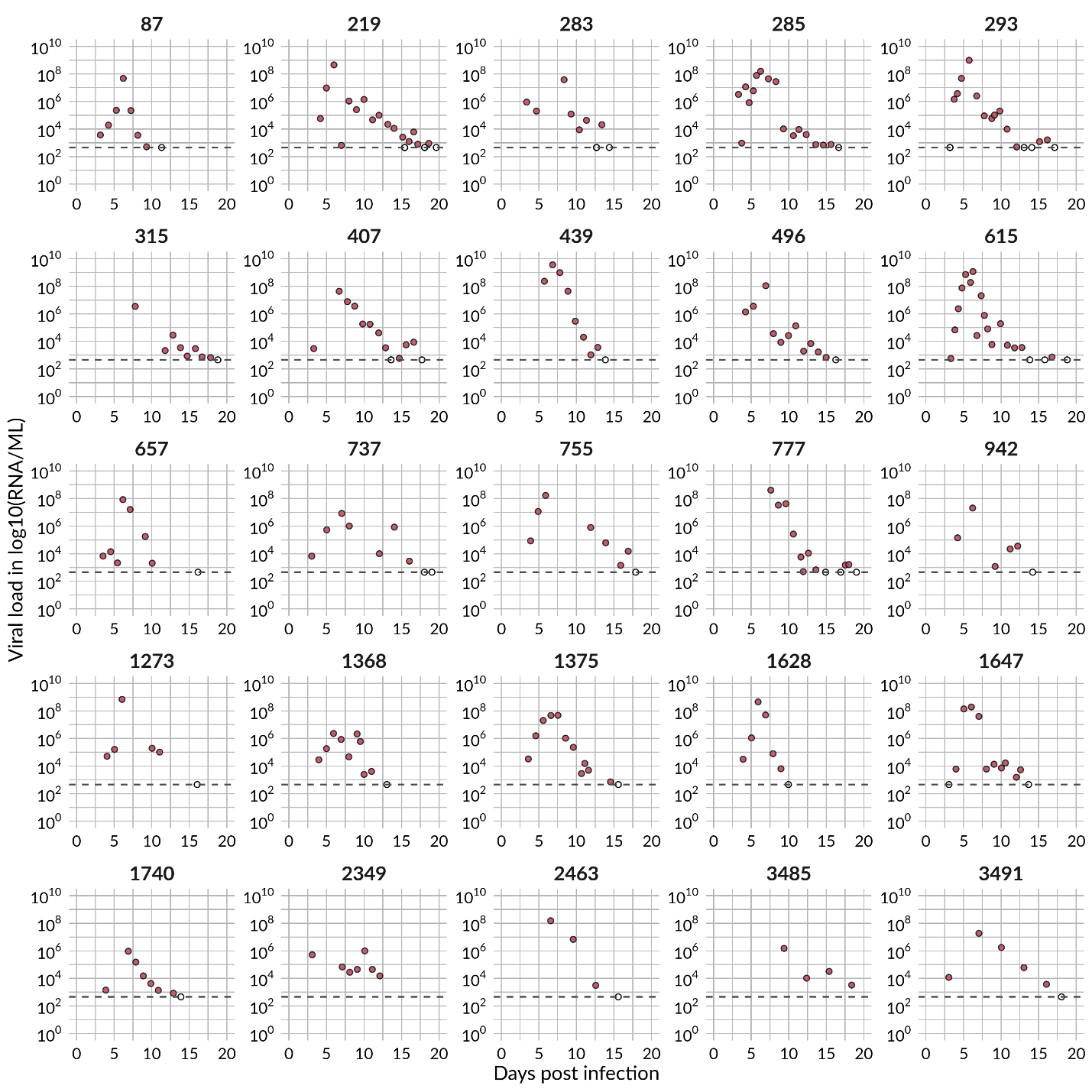


**Fig C: Data subset where data collection starts 3 days post infection.** Filled circles are measurement points, and non-filled circles are censored and below the detection limit (dotted grey line).

***Data subset where data collection starts 5 days post infection***
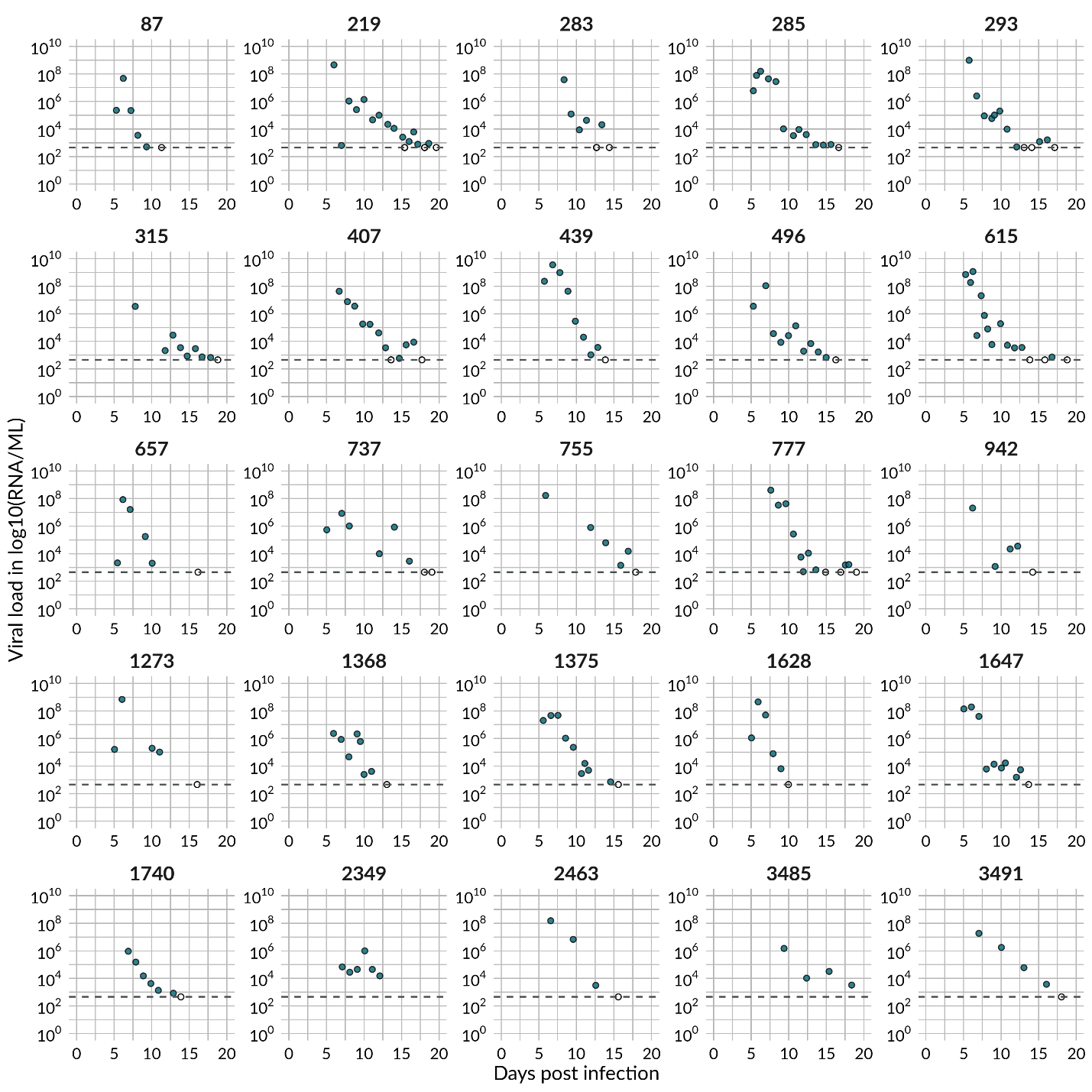


**Fig D: Data subset where data collection starts 5 days post infection.** Filled circles are measurement points, and non-filled circles are censored and below the detection limit (dotted grey line).

***Data subset where data collection starts 7 days post infection***
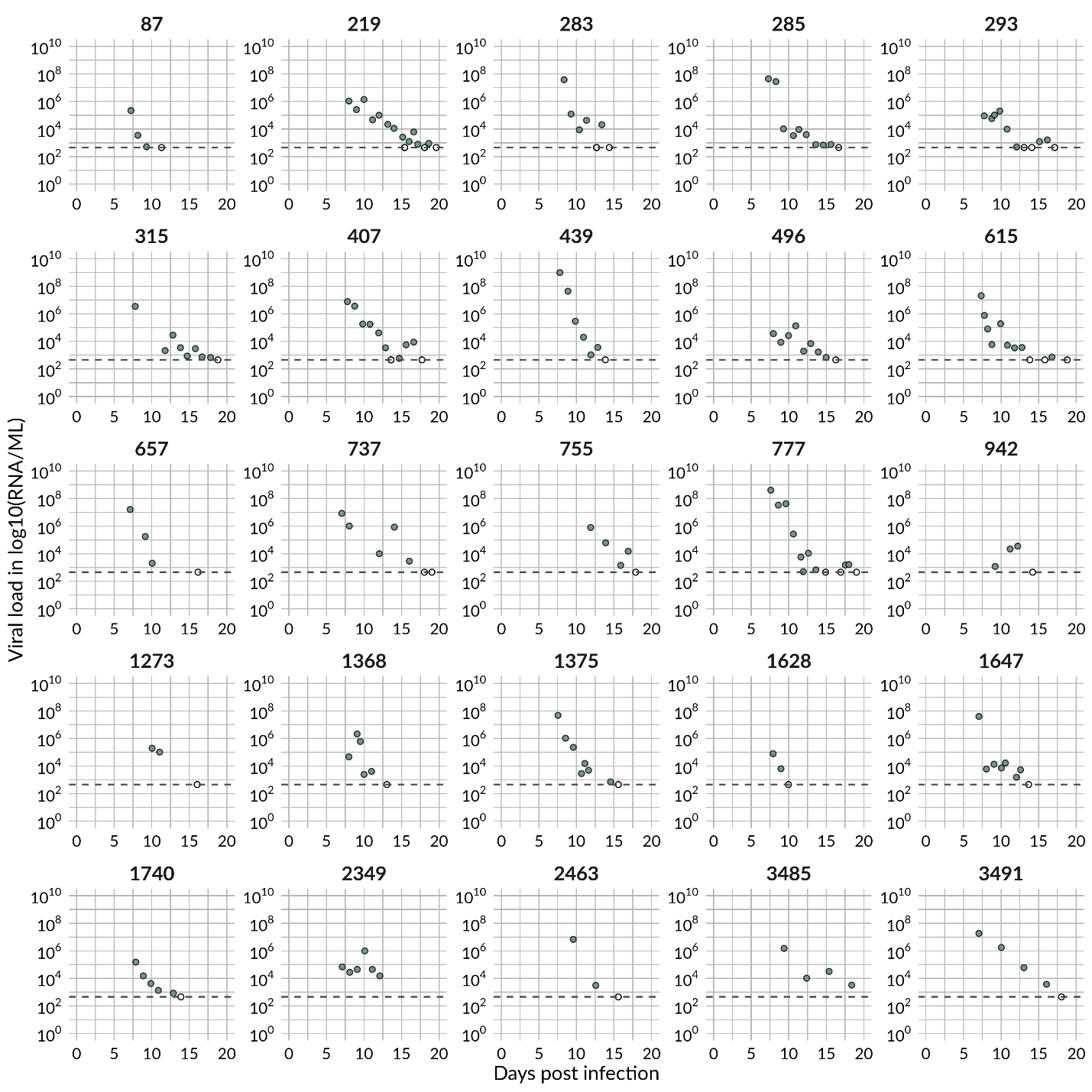


**Fig E: Data subset where data collection starts 7 days post infection.** Filled circles are measurement points, and non-filled circles are censored and below the detection limit (dotted grey line).

***Data subset where data collection starts post peak viral load***
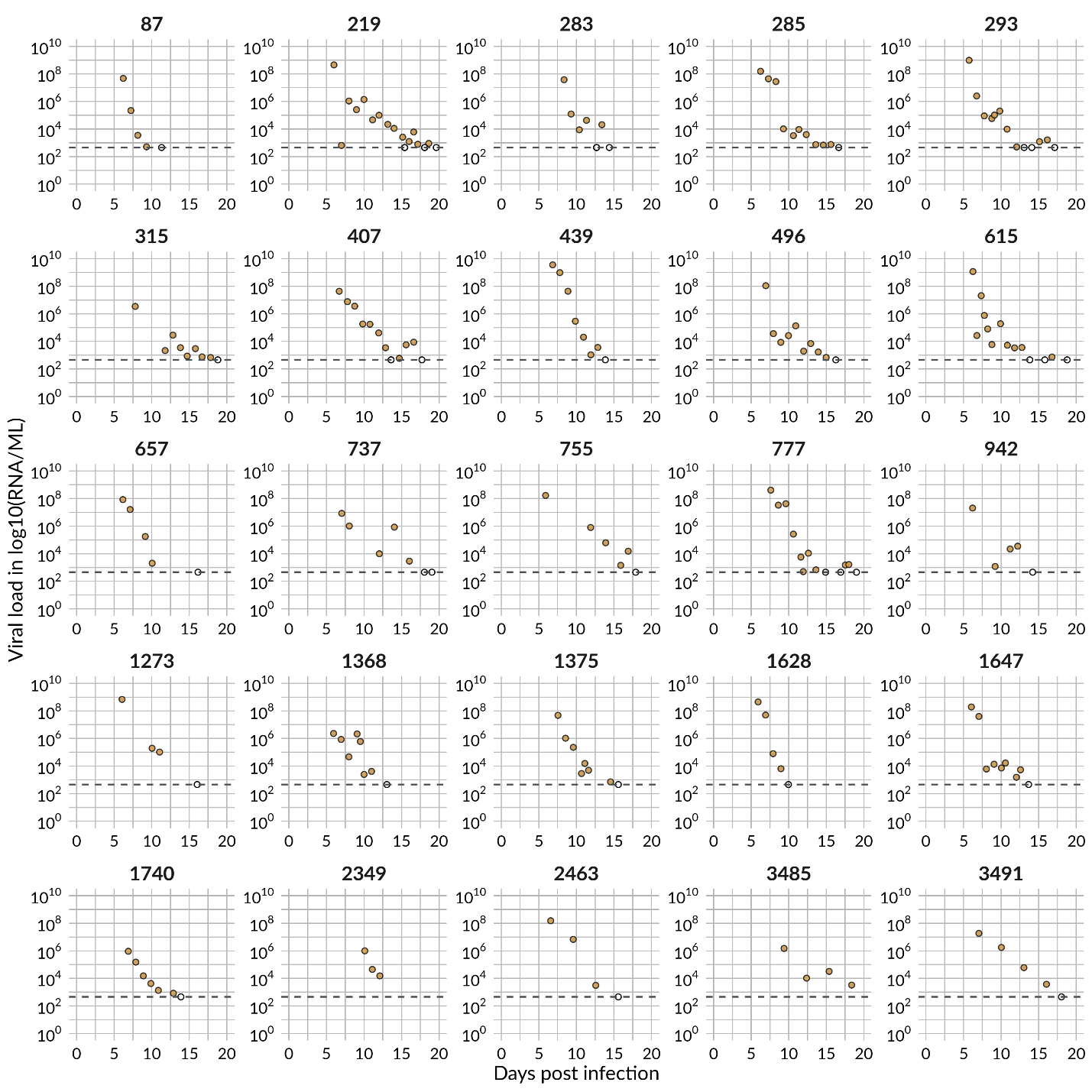


**Fig F: Data subset where data collection starts post peak viral load.** Filled circles are measurement points, and non-filled circles are censored and below the detection limit (dotted grey line).

# Profile likelihood estimation

Practical identifiability for the fitting of the full data set was analyzed using the profile likelihood estimation (PLE) method for each fitted model parameter using Monolix.

***Upper panel:*** For each parameter, the upper panel shows the profile of the analyzed parameter of interest. The model was re-fitted to the full data set keeping the parameter in each panel fixed at the values indicated on the x-axis. Note that for the PLE, random effects for the analyzed parameter of interest were disabled. The difference in resulting log-likelihood relative to the log-likelihood of the best model fit is plotted on the y-axis. The solid line indicates 5.99 which is the threshold for 95% confidence interval determined using a $\chi^{2}$ distribution with two degrees of freedom since in the full model both the population parameter and its random effects are estimated. A parameter is identifiable if its 95% confidence interval is finite. The dashed red line indicates the estimated model parameter.

***Lower panel:*** The lower panel shows how the remaining model parameters, which were re-estimated by re-fitting the model, change with the changing parameter of interest. The solid line indicates the trend of re-estimated model parameters. Fixed parameter values of the parameter of interest are plotted on the x-axis, while parameter values of re-estimated values are plotted on the y-axis.

For more information on PLE, see [3–5]

***
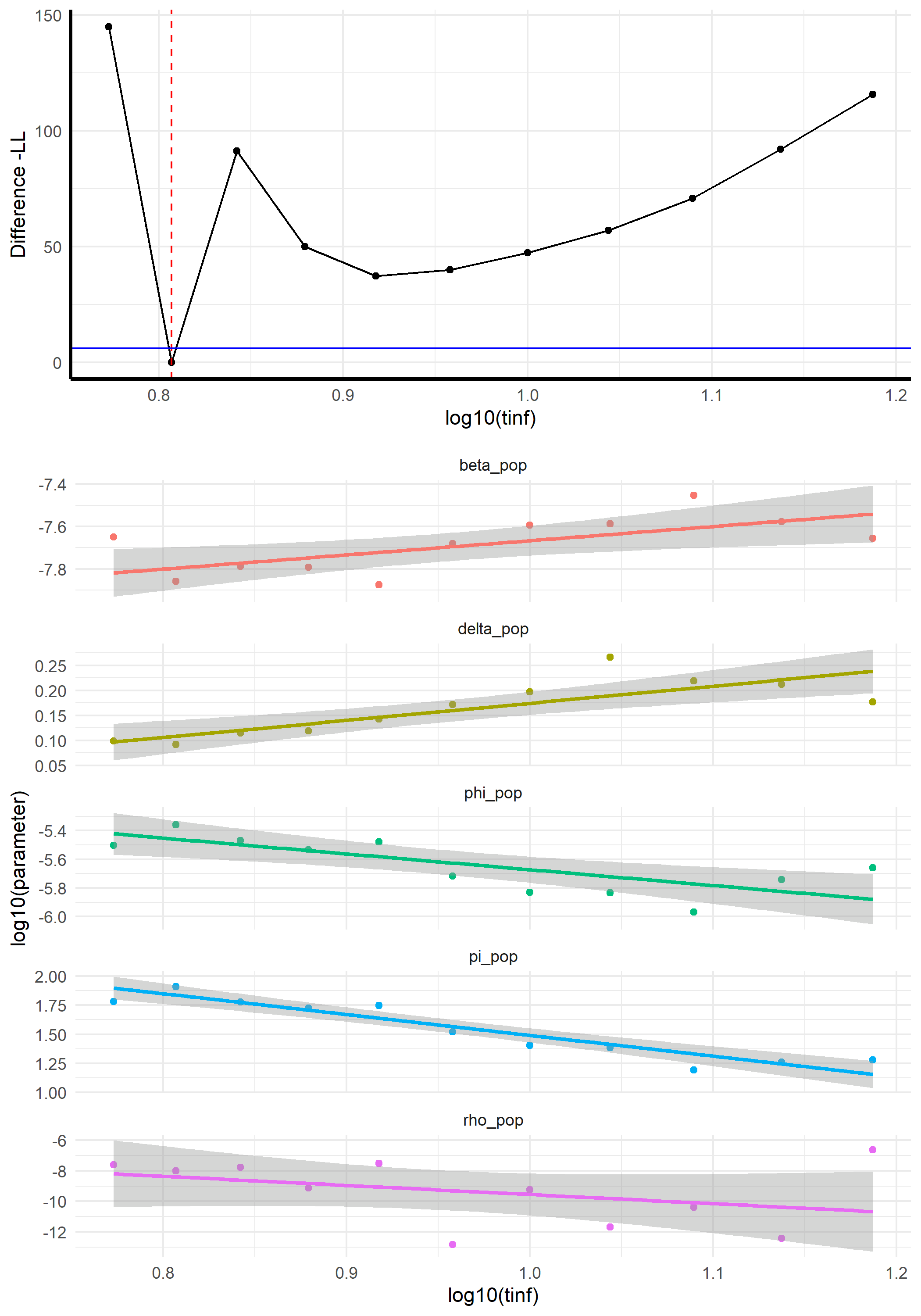
***

**Fig G: Parameter profile of the population parameter t_inf_**.

***
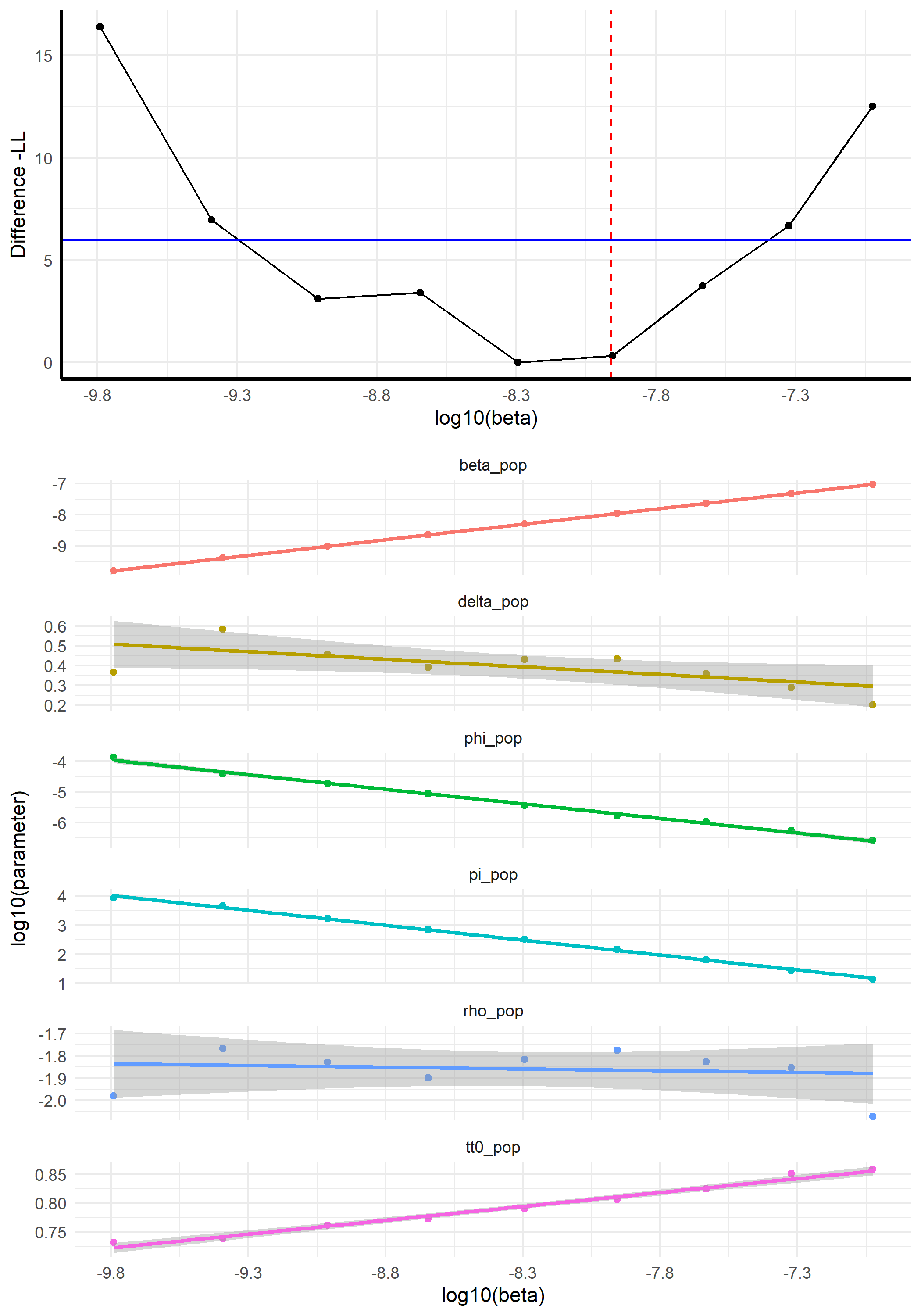
***

**Fig H: Parameter profile of the population parameter** $\boldsymbol{\beta}$**.**

***
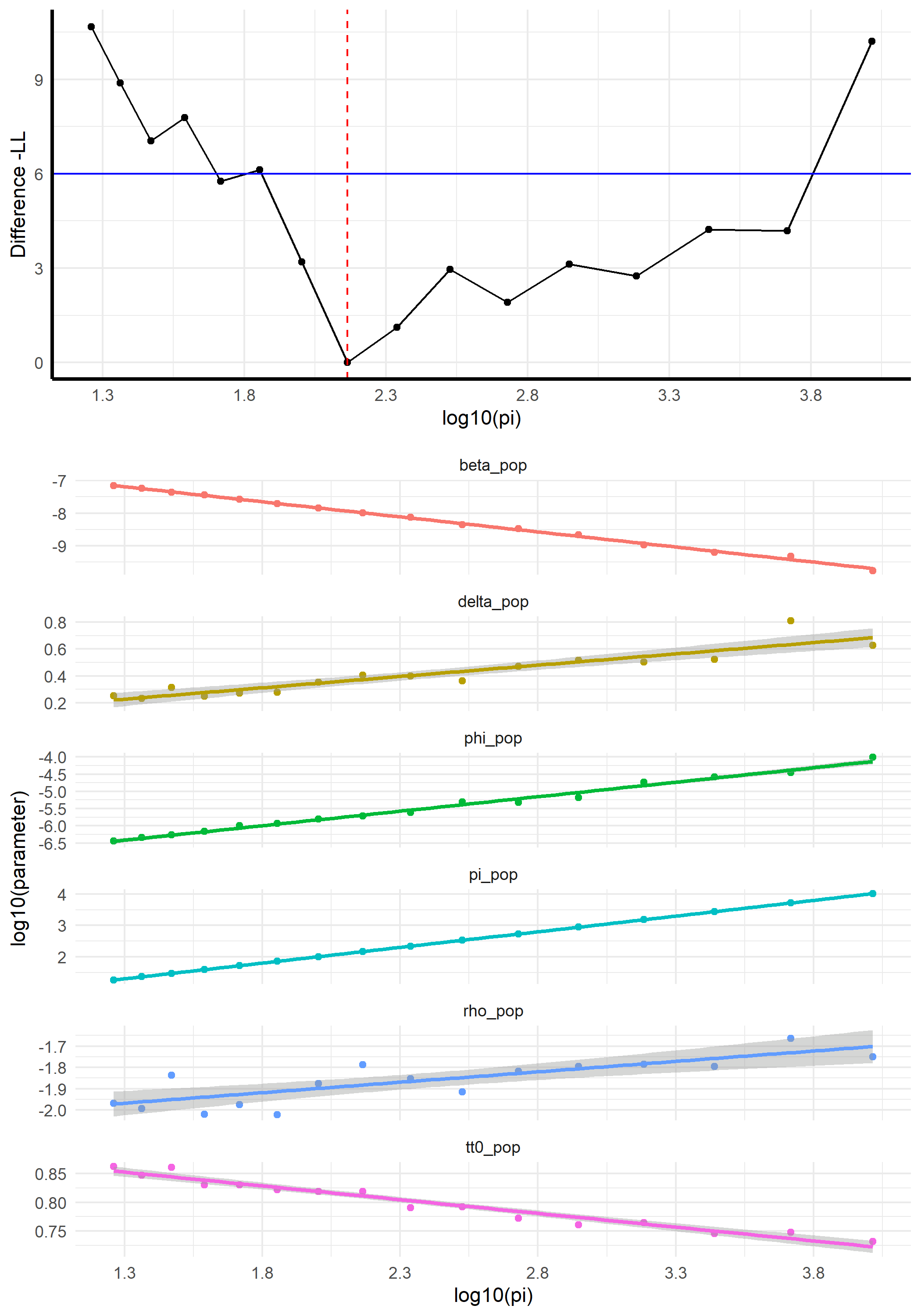
***

**Fig I: Parameter profile of the population parameter** $\boldsymbol{\pi}$**.**

***
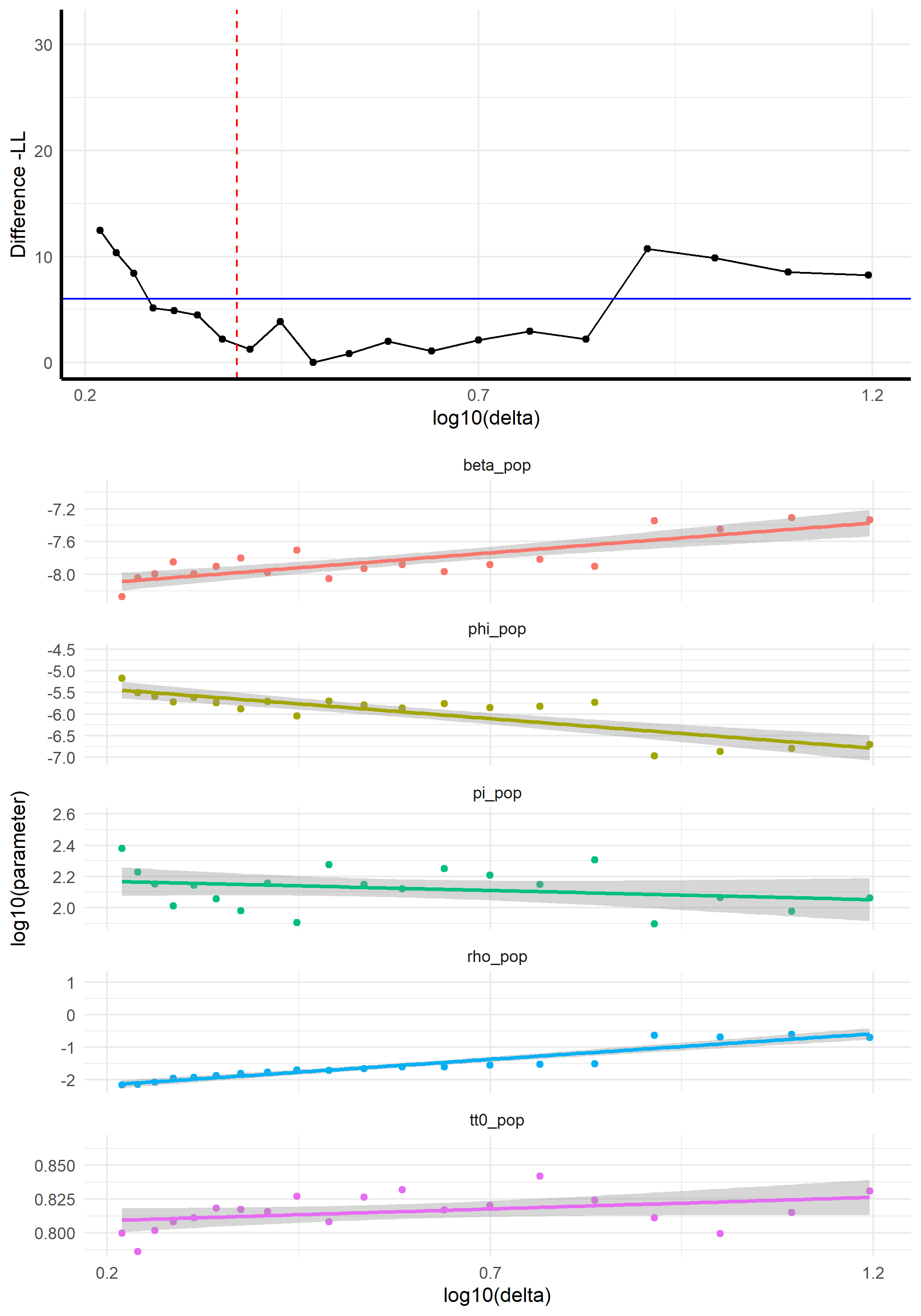
***

**Fig J: Parameter profile of the population parameter** $\boldsymbol{\delta}$**.**

***
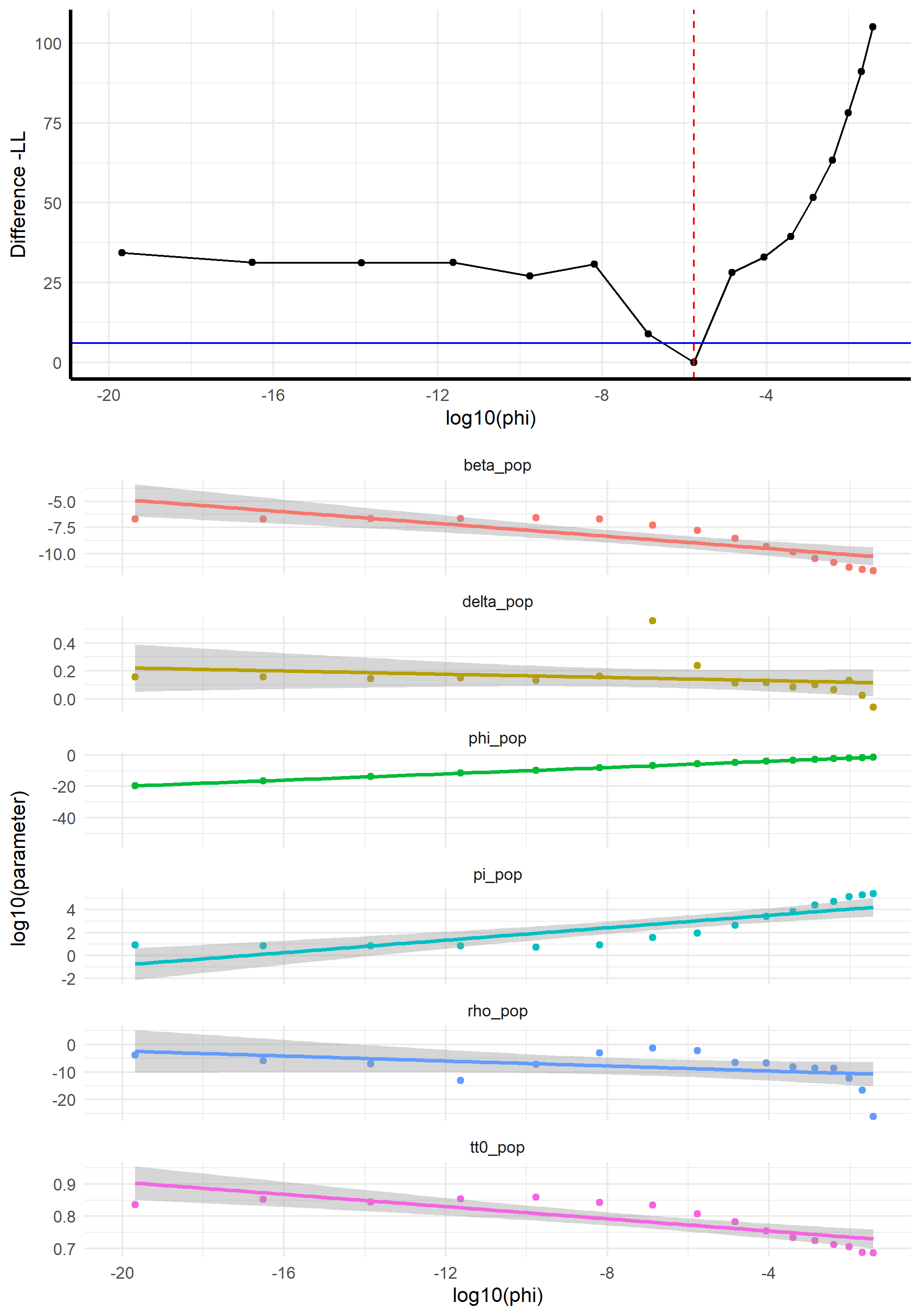
***

**Fig K: Parameter profile of the population parameter** $\boldsymbol{\phi}$**.**

***
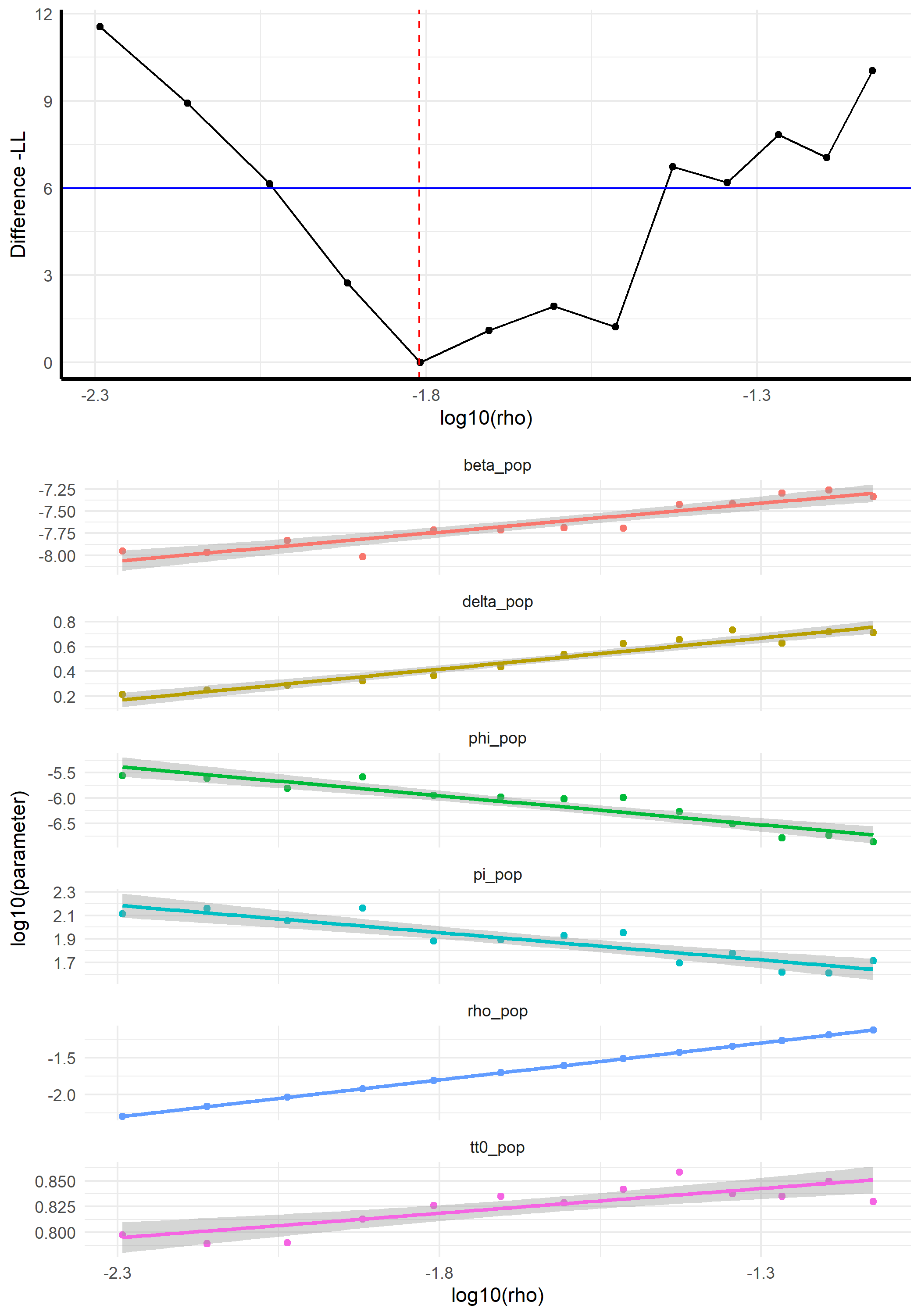
***

**Fig L: Parameter profile of the population parameter** $\boldsymbol{\rho}$**.**

# Estimated beta and pi values from the literature

***Table C: Estimated beta and pi parameter values from the literature.*** *TCLM = Target cell limited model, RCM = Refractory cell model*

*For plots, please see github.com/Carolin1901/SARS-CoV-2_parameter_reliability*

| ***Paper*** | ***Model*** | ***Figure 7 A*** | | | ***Figure 7 B*** | | | ***Figure 7 C*** | | |
| --- | --- | --- | --- | --- | --- | --- | --- | --- | --- | --- |
|  |  | $\boldsymbol{\beta}$***fixed*** | $\boldsymbol{\pi}$ ***fixed*** | ***RMSE*** | $\boldsymbol{\beta}$***fixed*** | $\boldsymbol{\pi}$ ***estimated*** | ***RMSE*** | $\boldsymbol{\beta}$ ***estimated*** | $\boldsymbol{\pi}$ ***fixed*** | ***RMSE*** |
| This  paper | RCM | 1.7 x 10^-8^ | 151 | 37 | 1.7 x 10^-8^ | 151 | 37 | 1.7 x 10^-8^ | 151 | 37 |
| [6] | TCLM | 3.2 x 10^-8^ | 4 | 84 | 3.2 x 10^-8^ | 97 | 43 | 7.9 x 10^-7^ | 4 | 42 |
| [7] | TCLM | 5.9 x 10^-8^ | 389 | 53 | 5.9 x 10^-8^ | 92 | 43 | 1.6 x 10^-8^ | 389 | 44 |
| [8] | TCLM | 5.2 x 10^-6^ | 4 | 45 | 5.2 x 10^-6^ | 71 | 53 | 7.9 x 10^-7^ | 4 | 42 |
| [9] | TCLM | 2.22 x 10^-8^ | 7280 | 54 | 2.22 x 10^-8^ | 174 | 45 | 1.6 x 10^-9^ | 7280 | 47 |
| [10] | RCM | 3.2 x 10^-8^ | 45 | 37 | 3.2 x 10^-8^ | 97 | 43 | 3.2 x 10^-7^ | 45 | 48 |
| [11] | RCM | 4.5 x 10^-9^ | 376 | 38 | 4.5 x 10^-9^ | 1003 | 45 | 6.3 x 10^-8^ | 376 | 53 |
| [12] | TCLM | 1.6 x 10^-9^ | 1710 | 35 | 1.6 x 10^-9^ | 3193 | 40 | 7.9 x 10^-8^ | 1710 | 55 |
| [13] | TCLM | 8.0 x 10^-6^ | 4 | 45 | 8.0 x 10^-6^ | 57 | 52 | 7.9 x 10^-7^ | 4 | 42 |

# References

1. Kissler SM, Fauver JR, Mack C, Tai CG, Breban MI, Watkins AE, et al. Viral dynamics of SARS-CoV-2 variants in vaccinated and unvaccinated persons. New England Journal of Medicine. 2021;385: 2489–2491. doi:10.1056/nejmc2102507

2. Kissler SM, Fauver JR, Mack C, Olesen SW, Tai C, Shiue KY, et al. Viral dynamics of acute SARS-CoV-2 infection and applications to diagnostic and public health strategies. PLoS Biol. 2021;19: e3001333. doi:10.1371/JOURNAL.PBIO.3001333

3. Kreutz C, Raue A, Kaschek D, Timmer J. Profile likelihood in systems biology. FEBS Journal. 2013. pp. 2564–2571. doi:10.1111/febs.12276

4. Raue A, Kreutz C, Maiwald T, Bachmann J, Schilling M, Klingmüller U, et al. Structural and practical identifiability analysis of partially observed dynamical models by exploiting the profile likelihood. Bioinformatics. 2009;25: 1923–1929. doi:10.1093/bioinformatics/btp358

5. Maiwald T, Hass H, Steiert B, Vanlier J, Engesser R, Raue A, et al. Driving the model to its limit: Profile likelihood based model reduction. 2016 [cited 3 Feb 2023]. doi:10.1371/journal.pone.0162366

6. Hernandez-Vargas EA, Velasco-Hernandez JX. In-host Mathematical Modelling of COVID-19 in Humans. Annu Rev Control. 2020;50: 448–456. doi:10.1016/J.ARCONTROL.2020.09.006

7. Goyal A, Reeves DB, Fabian Cardozo-Ojeda E, Schiffer JT, Mayer BT. Viral load and contact heterogeneity predict sars-cov-2 transmission and super-spreading events. Elife. 2021;10: 1–63. doi:10.7554/ELIFE.63537

8. Kim KS, Ejima K, Iwanami S, Fujita Y, Ohashi H, Koizumi Y, et al. A quantitative model used to compare within-host SARS-CoV-2, MERS-CoV, and SARS-CoV dynamics provides insights into the pathogenesis and treatment of SARS-CoV-2. PLoS Biol. 2021;19: e3001128. doi:10.1371/journal.pbio.3001128

9. Padmanabhan P, Desikan R, Dixit NM. Modeling how antibody responses may determine the efficacy of COVID-19 vaccines. Nature Computational Science 2022 2:2. 2022;2: 123–131. doi:10.1038/s43588-022-00198-0

10. Ke R, Zitzmann C, Ho DD, Ribeiro RM, Perelson AS. In vivo kinetics of SARS-CoV-2 infection and its relationship with a person’s infectiousness. Proc Natl Acad Sci U S A. 2021;118. doi:10.1073/PNAS.2111477118/-/DCSUPPLEMENTAL

11. Ke R, Martinez PP, Smith RL, Gibson LL, Mirza A, Conte M, et al. Daily longitudinal sampling of SARS-CoV-2 infection reveals substantial heterogeneity in infectiousness. Nature Microbiology 2022 7:5. 2022;7: 640–652. doi:10.1038/s41564-022-01105-z

12. Perelson AS, Ribeiro RM, Phan T. An explanation for SARS-CoV-2 rebound after Paxlovid treatment. medRxiv. 2023; 2023.05.30.23290747. doi:10.1101/2023.05.30.23290747

13. Ejima K, Kim KS, Ludema C, Bento AI, Iwanami S, Fujita Y, et al. Estimation of the incubation period of COVID-19 using viral load data. Epidemics. 2021;35: 100454. doi:10.1016/J.EPIDEM.2021.100454
